# Sequences From First Settlers Reveal Rapid Evolution in Icelandic mtDNA Pool

**DOI:** 10.1371/journal.pgen.1000343

**Published:** 2009-01-16

**Authors:** Agnar Helgason, Carles Lalueza-Fox, Shyamali Ghosh, Sigrún Sigurðardóttir, Maria Lourdes Sampietro, Elena Gigli, Adam Baker, Jaume Bertranpetit, Lilja Árnadóttir, Unnur Þorsteinsdottir, Kári Stefánsson

**Affiliations:** 1 deCODE Genetics, Reykjavik, Iceland; 2 Department of Anthropology, University of Iceland, Reykjavik, Iceland; 3 Institute of Evolutionary Biology (UPF-CSIC), Barcelona, Spain; 4 Department of Human Genetics, Leiden University Medical Centre, Leiden, The Netherlands; 5 National Museum of Iceland, Reykjavik, Iceland; 6 Faculty of Medicine, University of Iceland, Reykjavik, Iceland; University of Oxford, United Kingdom

## Abstract

A major task in human genetics is to understand the nature of the evolutionary processes that have shaped the gene pools of contemporary populations. Ancient DNA studies have great potential to shed light on the evolution of populations because they provide the opportunity to sample from the same population at different points in time. Here, we show that a sample of mitochondrial DNA (mtDNA) control region sequences from 68 early medieval Icelandic skeletal remains is more closely related to sequences from contemporary inhabitants of Scotland, Ireland, and Scandinavia than to those from the modern Icelandic population. Due to a faster rate of genetic drift in the Icelandic mtDNA pool during the last 1,100 years, the sequences carried by the first settlers were better preserved in their ancestral gene pools than among their descendants in Iceland. These results demonstrate the inferential power gained in ancient DNA studies through the application of population genetics analyses to relatively large samples.

## Author Summary

Ancient DNA studies have great potential to shed light on the evolution of populations because they provide the opportunity to sample from the same population at different points in time. However, ancient DNA studies are often based on DNA extracted from only one or a few individuals and, therefore, do not lend themselves to statistical inference. Here, we describe the analysis of a sample of mitochondrial DNA (mtDNA) control region sequences from 68 Icelandic skeletal remains that are about 1,000 years old, from the time that Iceland was first settled. We show that the ancient Icelandic mtDNA sequences are more closely related to sequences from contemporary inhabitants of Scotland, Ireland, and Scandinavia (and several other European populations) than to those from the modern Icelandic population. It appears that the array of sequences carried by the first generations of Icelanders was better preserved in the gene pools of their ancestors than among their modern descendants because of a faster rate of evolution due to genetic drift in the Icelandic mtDNA pool during the last 1,100 years. These results demonstrate the inferential power that can be gained from studies by applying the methods of population genetics to samples of ancient DNA sequences.

## Introduction

**Figure 1:**
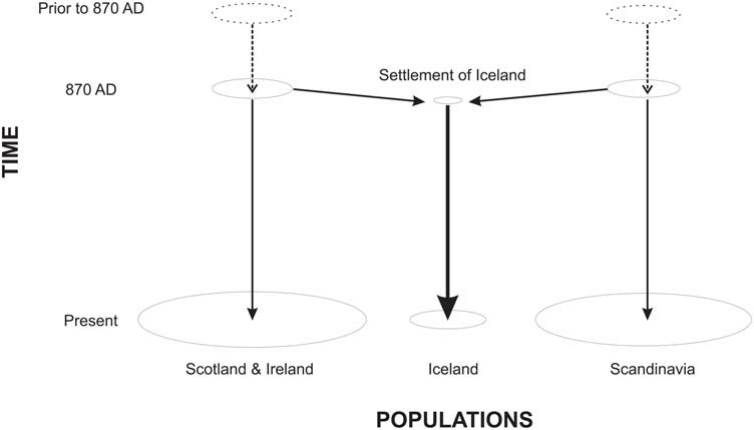
A schematic representation of Iceland's population history. The circles represent the gene pools of the Scottish and Irish, Icelandic and Scandinavian population groups at different points in time. Circle diameter broadly reflects the relative population sizes. The vertical arrows represent the transmission of DNA between generations within populations, while the diagonal arrows represent the settlement of Iceland from 870 to 930 AD from Scotland, Ireland and Scandinavia.

The Icelanders are one of the most studied populations in human genetics [1]–[5]. According to historical and archaeological sources, Iceland was settled roughly 1100 years ago by a mixture of people that originated primarily from Scandinavia and the British Isles [6],[7]. Studies of mtDNA variation indicate that contemporary Icelanders trace about 37% of their matrilineal ancestry to Scandinavia, with the remainder coming from the populations of Scotland and Ireland [1],[8],[9]. In contrast, Y-chromosome analyses suggest that 75–80% of their patrilineal ancestry originated in Scandinavia [3],[9]. Like most admixture estimates in the literature, these were based on samples from contemporary populations rather than samples from the period of the admixture event (Figure 1). As each gene pool was affected by a combination of evolutionary forces during the intervening time, the ancestry of the admixed population may have been considerably altered at any particular locus. Given that only about 35 generations have passed since the settlement of Iceland, it is unlikely that its gene pool has diverged notably due to the accumulation of mutations, and there is no evidence for large-scale immigration after the initial settlement period. However, there is evidence for relatively high levels of genetic drift in this population due to its small size [2],[10].

**Figure 2:**
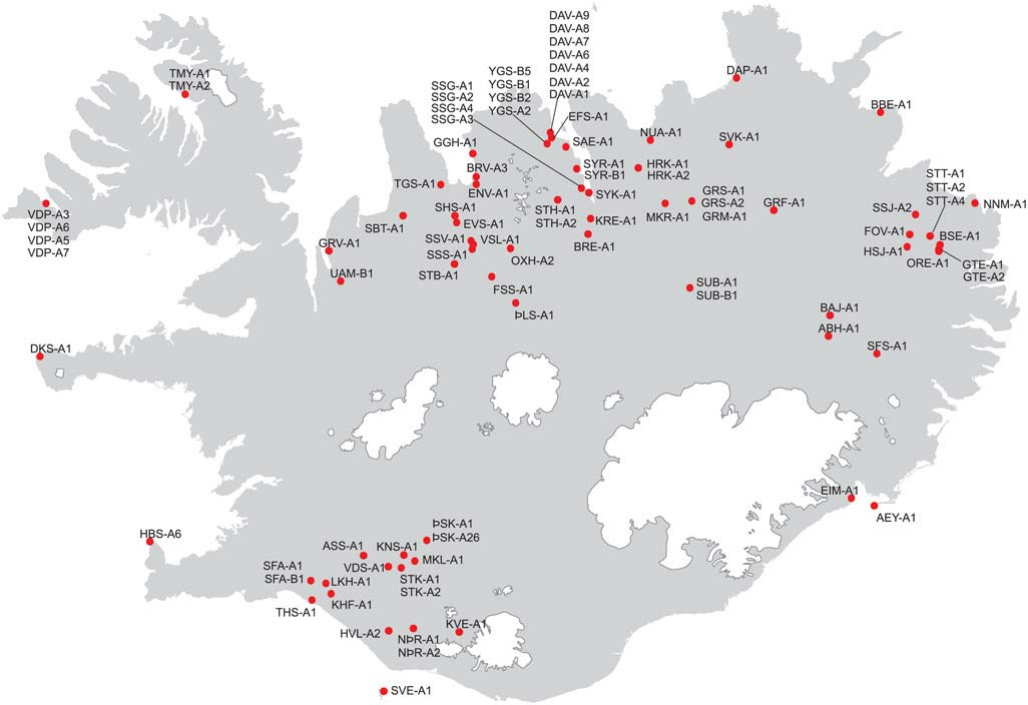
The geographical distribution of sampled skeletal remains in Iceland. The figure shows a map of Iceland, with the locations of 67 excavation sites represented by red dots. The code names of 95 skeletal remains are shown next to the sites from which they were retrieved.

Several previous ancient DNA studies have addressed the question of evolutionary change within species or populations, but none with a sample size sufficient to detect changes over a short time-scale [11]–[16]. In order to directly assess the magnitude of evolutionary change in the Icelandic mtDNA pool and to estimate the matrilineal ancestry of the population at the time of settlement, we extracted DNA from 98 teeth obtained from 95 skeletal remains of individuals thought to have died in Iceland before 1000 AD or soon after and excavated from 1880 to 1996 (Figure 2). An attempt was made, for each sample, to amplify, clone and sequence 743 nucleotides from the mtDNA control region (spanning sites 16055-16410 and 16517-334) in six different fragments. Using a multifaceted quality control procedure, it was possible to successfully assign full sequences to 73 of the 95 skeletal remains. Support for the authenticity of these sequences was provided by meta-analyses of secondary variables such as excavation date, sequence length and contamination levels. Statistical tests from population genetics were then used to assess the relationship between the sample of mtDNA sequences from the skeletal remains and those from several contemporary populations. The results demonstrate that the ancient Icelandic mtDNA sequences are more closely related to sequences from present inhabitants of Scotland, Ireland and Scandinavia (and several other European populations), than to those from their descendants, the modern Icelandic population. We propose that this is due to a faster rate of genetic drift in the Icelandic mtDNA pool during the last 1100 years, than in the mtDNA pools of the larger European populations.

## Results

### Amplification, Cloning, and Sequencing

For each of the 97 DNA extractions performed in Iceland, an attempt was made to obtain at least one positive amplification for each of the six fragments (16055-16218, 16209-16410, 16055-16410, 16517-160, 183-409/183-334 and 16517-409/16517-334). If a positive amplification was not obtained in the first attempt, and a positive amplification had not been obtained for an overlapping fragment for the DNA extract in question, then at least one more attempt was made. In total, 1239 PCRs were performed using the 97 DNA extracts from 95 different sampled individuals, of which 860 yielded visible bands that were cut from gels and stored for further analysis, representing an average of 1.48 successful amplifications per fragment per DNA extract.

A total of 532 of the 860 successful amplifications were selected for cloning and sequencing, with primary emphasis placed on obtaining sequence data from at least the four shorter fragments for each of the skeletal remains. In 33 cases, two different amplifications of the same fragment from the same DNA extract were cloned for the purpose of quality control (one fragment was cloned from three different amplifications). In 10 cases, amplifications of the same fragment from different DNA extracts of the same skeletal remains were cloned. In all, the 532 cloned amplifications represent 487 distinct fragments from the 95 skeletal remains.

Where possible at least 12 colonies carrying cloned PCR products were selected for sequencing from each culture of transformed bacteria. In cases where no colonies carrying cloned PCR products were obtained from a culture of transformed bacteria, we repeated the cloning experiment with a different amplification of the same fragment. If a different amplification was not available, the cloning experiment was repeated with a second PCR performed using the original amplification as template DNA and the same pair of primers.

At least one clone sequence was obtained from 448 of the 492 distinct fragments subjected to cloning from the 95 different skeletal remains (corresponding to 456 of 502 distinct fragments from the 97 DNA extracts). Both strands were sequenced for all harvested clones, yielding complete sequences for a total of 5037 clones (an average of 11.24 clone sequences per fragment per skeletal remain). Table S1 shows a breakdown of the number of experiments performed per fragment per sample along with the number of successful amplifications and the number of clone sequences obtained. The full set of clone sequences is provided in Table S2.

Clone sequences were obtained from separate amplifications from the same DNA extract for 25 fragments. For nine additional fragments, clone sequences were obtained from separate amplifications of DNA extracted from different teeth of the same skeletal remains (samples HSJ-A1 and SSG-A1). This redundancy was exploited to assess the reliability of sequence assignment to samples.

### Assignment of mtDNA Sequences to Skeletal Remains

A total of 280 of the 448 fragments (62.5%) yielded a maximum value of the *c*-statistic (*c_MAX_*) that was significantly greater than the expectation based on the null-hypothesis (P<0.05). The finding of a signal of mutational differences attributable to cytosine deamination in the majority of fragments suggests that old template molecules were present in the DNA extracts. The most straightforward interpretation of this finding is that they represent endogenous DNA template molecules. Although contaminant template molecules may also carry damage due to cytosine deamination [17], it is very unlikely that the magnitude of contamination from a single source per DNA extract would be sufficient to produce a signal detectable with the *c*-statistic.

The true proportion of fragments exhibiting a signal of damage due to cytosine deamination is likely to be considerably greater than 62.5%, because a small number of clone sequences obtained for some fragments reduced the power to detect this signal of damage. Thus, when only fragments with more than five clone sequences were examined, 270 of 386 (69.95%) yielded a statistically significant c_MAX_ value and when the threshold is set to 10 clone sequences or more, 227 of 300 (75.67%) c_MAX_ values were statistically significant.

A total of 19 samples yielded candidate sequences for all six fragments. Of these, six showed perfect compatibility between candidate sequences according to the evaluation of overlapping fragments and haplogroup assignment (FSS-A1, GRM-A1, HSJ-A1, KNS-A1, MKR-A1 and TGS-A1). With relaxed matching conditions of overlapping fragments, an additional four samples with compatible candidate sequences for all fragments were identified (ASS-A1, DKS-A1, GRF-A1 and SSG-A1). Specifically, these conditions were applied to six fragments in the four samples. In each case, incompatibility was attributable to a single mutation consistent with being due to a cytosine deamination event and the sequence without the mutation was present among the other clone sequences for the fragment with a statistically significant value of the c-statistic (the exception was fragment 16517-409 in sample DKS-A1, which yielded only three clone sequences).

In the case of the ten samples for which compatible candidate sequences were obtained for all fragments, the sequence assignment procedure was considered to have been completed successfully. The remaining 85 samples were subjected to the qualitative assessment procedure described in the materials and methods section. This resulted in the selection of an alternative candidate sequence for 76 fragments (out of the 388 fragments from these samples which yielded clone sequences). The incompatibilities or unlikely mutation combinations resolved by these 76 changes can be broken down as follows. About half, or 37, were attributed to cytosine deamination events, ten to differences at highly polymorphic indel sites (primarily sites 16183 and 302), ten to presumed jumping PCR or PCR misincorporation events and, finally, 19 were attributed to contaminant sequences (an assumption based on there being a pattern of mutational differences between the initial and alternative candidate sequences that did not fit any of the previous categories and the observation that the initial candidate sequence was relatively common in contemporary human populations). Eleven fragments were disregarded from further analysis, as the available clone sequences both did not match candidate sequences from overlapping fragments from the same sample and had haplogroup assignments that were incompatible with the candidate sequences from other fragments. In all cases, the disregarded fragments were from amplifications of the longer sequence regions, 16055-16410 or 16517-334/409, which are less likely to yield sequences derived from endogenous template molecules than the shorter regions. Information about the initial candidate sequences proposed for each of the 448 fragments on the basis of the *c*-statistic and the changes made as a result of the qualitative assessment procedure is provided in Table S3.

In addition to the validation of candidate sequences through the amplification of overlapping fragments, confirmation was sought through independent amplifications of the same sample for a subset of fragments. In the Reykjavik lab, two independent amplifications from the same DNA extract were performed for 24 fragments from 17 samples. In 21 instances, the candidate sequence was observed among the clone sequences obtained from both amplifications. The three exceptions were samples BSE-A1, ASS-A1 and SSG-A4, in all cases for fragment 16055-16410, where the candidate sequence was seen in only one of the amplifications. Amplifications from separate DNA extracts were also performed in the Reykjavik lab for nine fragments from two samples. In each case, the candidate sequence was present in both amplifications (Table S4). Finally, clone sequences were obtained for 18 fragments from 12 DNA extractions performed in the Barcelona lab (thereof two from different teeth of the same sample, FOV-A1). Sixteen of the fragments yielded clone sequences that matched the candidate sequence identified on the basis of the DNA extractions performed in Reykjavik. The two fragments that did not replicate were both from the same sample (STK-A1). An overview of the results from the replication amplifications performed in Barcelona is presented in Table S5.

The final step was to determine for which of the 85 samples, processed as described above, the sequence assignment procedure could be considered as successfully completed. Several criteria were applied to this end. First, it was required that clone sequences were available for combinations of fragments that spanned the regions 16055-16410 and 16517-334. Twelve samples did not meet this criterion (ABH-A1, AEY-A1, BRV-A3, DAV-A2, DAV-A6, GTE-A2, HRK-A1, HVL-A2, STT-A1, THS-A1, VSL-A1 and YGS-B5) and were therefore excluded from further analysis. Second, it was required that samples had no unresolved incompatibilities between fragments. Six samples did not meet this criterion (SAE-A1, KVE-A1, TMY-A1, SFA-A1, DAV-A4 and TMY-A2) and were excluded from further analysis.

Seventeen samples met the criteria of having candidate sequences completely spanning both the studied regions with compatible haplogroup assignments, but did not provide candidate sequences from any fragments that overlapped. Due to the greater uncertainty this engendered about the sequence assignment for these samples, the combinations of mutations from different fragments were screened against phylogenetic networks of European mtDNA sequences from the literature [8],[18],[19]. A perfect match for each sample with sequences in the published networks was not required. Rather, the aim was to identify unusual combinations of mutations that could be the result of contamination in one or more of the fragments from a particular sample. Two of the 17 samples were flagged in this assessment. Sample STH-A2 because of the combination of the motif 16298C (16055-16410) and the mutation 73G, and sample STT-A4 because of the combination of motif 16167T (16055-16218) and motifs 16343G 16390A (16209-16410) and 16519C 73G 150T (16517-160). While these combinations of mutations cannot be ruled out, they are unlikely given what is known about the phylogeny of European mtDNA sequences and the two samples were therefore excluded from further analysis. The remaining 15 samples (BSE-A1, EFS-A1, EIM-A1, ENV-A1, GRV-A1, KHF-A1, NÞR-A1, OXH-A2, SSG-A2, SUB-B1, SYR-A1, SYR-B1, VDS-A1, ÞSK-A26 and YGS-A2) were deemed to have combinations of mutations that were congruent with the known phylogeny of European mtDNA sequences.

Finally, the sample STK-A1 was excluded on the basis of strong evidence for pervasive contamination from the researcher (A.H.) involved in almost all the pre-PCR laboratory work performed in Reykjavik. Thus, all five fragments from this sample that yielded clone sequences were consistent with the haplogroup H sequence of A.H. (16055-16410: 16129A 16239T and 16517-409: 16519C 152C 263G 315_1C) and none of the fragments showed any signal of cytosine deamination damage. Moreover, as previously noted, a DNA extraction performed in the Barcelona lab, yielded completely different sequences for two fragments (16209-16401: 16224C 16311C 16320T and 16517-160: 16519C 73G 146C 152C), both of which are consistent with membership in haplogroup K. These clone sequences yielded statistically significant values of the *c*-statistic, suggesting that they derive from the endogenous template molecules from the STK-A1 sample. Nonetheless, sample STK-A1 was excluded from further analysis, as the clone sequences did not fully span the regions 16055-16410 and 16517-334. Another sample, EFS-A1, was also excluded due to a suspicion that the clone sequences obtained for fragment 16209-16410 derived from contaminant templates from the researcher A.H.

In conclusion, sequence assignment was deemed to be unsuccessful for 22 skeletal remains, either because clone sequences could not be obtained for part of the control region (12 remains), because of incompatibility between candidate sequences from the different amplified fragments (8 remains) or suspicion of contamination (2 remains). These 22 remains were excluded from further analysis, whereas sequence assignment was deemed successful for 73 of the 95 skeletal remains.

### Meta-Analyses of Sequence Authenticity

In addition to overall age, one of the factors thought to affect the success of DNA analysis from ancient tissues is the time from excavation [20]–[22]. This may be particularly important when post-excavation storage conditions (i.e. room temperature and periodic human handling) are less favourable for DNA preservation than pre-excavation conditions (soil temperatures ranging 0–10°C [23]). Thus, as time passes after excavation, one would expect a reduction in the number of intact endogenous template molecules and an increase in the number of exogenous contaminant template DNA molecules extracted from a tissue sample. A t-test was performed to determine whether time from excavation played a role in the success rate of DNA analysis from the 95 skeletal remains. We observed a statistically significant difference (*P* = 0.000215) between the mean excavation year of the 73 successfully analysed remains (1950.5±2.47) and that of the 22 unsuccessfully analysed remains (1930.6±4.59). This indicates that the amount of analysable DNA in the teeth and the ability to obtain compatible sequences from different fragments diminishes with increasing time from excavation, which supports the authenticity of the candidate sequences from the successfully analysed samples (as the opposite would be expected if most of the analysable DNA was contamination).

We next assessed the correlation between the excavation year of samples and the degree of contamination in the clone sequence sets. For this purpose, the degree of contamination was defined as the proportion of clone sequences per amplified fragment per sample that were found in a reference database of mtDNA sequences from contemporary human populations. The expectation is a greater proportion of contaminant clone sequences with increasing time from excavation. Under the assumption that all database matches were due to contamination, Spearman's rank correlation coefficient (*ρ*) was −0.004 (*P* = 0.944), calculated for the 386 clone sequence sets with more than five clone sequences. In contrast, when database matches with the sequences postulated to represent the endogenous template molecules (the so-called candidate sequence, see materials and methods section) were not counted as contaminants, a statistically significant negative correlation between the degree of contamination and excavation year was observed, *ρ* = −0.259 (*P*<10^−6^). Similar results were observed when each of the six fragments was examined separately, with four fragments yielding P<0.05 (see Table 1), the probability of which is 0.000086 under a binomial distribution. These results are incompatible with the conservative hypothesis that the candidate sequences derive from contaminant templates, but are consistent with our proposition that they represent endogenous templates.

**Table 1:** The relationship between excavation year and the proportion of contaminant clone sequences.

		Proportion of contaminant clone sequences	Excavation Year^a^	Correlation	
Sequence fragment	N	Mean (S.E.)	Mean (S.E.)	Spearman's *ρ*	P-value
16055-16218	87	0.18 (0.021)	1946.5 (2.46)	−0.214	0.046
16055-16410	48	0.27 (0.042)	1948.8 (3.68)	−0.026	0.861
16209-16410	79	0.18 (0.024)	1947.1 (2.39)	−0.26	0.021
16517-160	85	0.17 (0.023)	1945.3 (2.5)	−0.505	<10−6
16517-334/409	19	0.26 (0.07)	1948 (5.74)	−0.533	0.019
183-334/409	68	0.19 (0.018)	1947.8 (2.54)	−0.113	0.36
All fragments	386	0.19 (0.011)	1946.9 (1.15)	−0.259	<10−6

a The range of excavation years was 1880 to 1996 for all sequence fragments, except for 16517-334/409 and 183-334/409, which were 1909 and 1901, respectively.

Table 1 indicates that clone sets from the two longest fragments have, on average, the greatest proportion of contaminant sequences. We assessed the relationship between fragment length and the proportion contaminant sequences through the correlation, for all pairs of clone sets obtained from the same sample, between the difference in fragment length and the difference in the proportion of contaminant sequences. This is an effective way to test for the correlation between contamination and sequence length, whilst controlling for the different background levels of contamination in samples. Analysis was restricted to clone sets with more than five sequences, which gave a total of 665 pairs of clone sets. When all clone sequences with reference database matches were counted as contaminant sequences, no correlation was seen (*ρ* = −0.003, *P* = 0.934). Conversely, when candidate sequences were not counted as contaminants, a significant positive correlation was seen between the difference in fragment length and the difference in the degree of contamination, *ρ* = 0.199 (*P*<10^−6^). As before, this result is consistent with the candidate sequences representing endogenous rather than contaminant template molecules.

### Population Genetic Analyses of the Early Medieval Icelandic mtDNA Sequences

The 73 sequences that were successfully assigned to skeletal remains, spanning sites 16055-16410 and 16517-334, were grouped into 58 mutationally distinct haplotypes (*k*), characterised by a gene diversity (*GD*) of 0.9897 (ignoring the unstable length polymorphisms associated with poly-C stretches between sites 302-315 and 16183-16193). In order to define a sample of early medieval Icelandic mtDNA sequences for comparative population analyses, we excluded two of the 73 skeletal remains because of unknown temporal provenance (TGS-A1 and SSS-A1). In addition, three pre-Christian skeletal remains (DAV-A9, YGS-B2 and SUB-B1) were excluded on the basis of having sequences that were identical to others from nearby graves at the same burial sites (DAV-A8, YGS-B1 and SUB-A1, respectively). These were the only instances in our data of skeletal remains suspected to be closely related through the female line from the pre-Christian excavation sites that are thought to have been family burial sites. This resulted in an Icelandic early medieval sample (IEMS) of 68 sequences with *k* = 57 and *GD* = 0.9934.

Previous analyses of the matrilineal ancestry of contemporary Icelanders have provided estimates of 62.5–66% admixture from Scotland and Ireland, with the remaining 34–37.5% coming from Scandinavia [1],[9]. Given the difference between the IEMS and contemporary Icelanders, it is of interest to determine whether a similar partitioning of ancestry is observed for the IEMS. To estimate admixture, we used a maximum likelihood estimator of the *mρ* statistic [3], with 1000 iterations of a bootstrap procedure that sampled with replacement from the admixed and source populations to provide 95% confidence intervals (C.I.). For each iteration of the bootstrap an equal number of sequences was sampled from the Scottish/Irish and Scandinavian source population samples to avoid the impact of sample size differences on the results (N = 898, the size of the smaller Scandinavian sample). Using the sequence data described in Table 1, we obtained an estimate of 58% ancestry from Scotland and Ireland for contemporary Icelanders (95% C.I.: 44.6–71.2%). In comparison, the IEMS yielded an estimate of 64.7% (95% C.I.: 36.8–90.3%), indicating a similar excess of matrilineal ancestry from Scotland and Ireland.

A comparison with a sample of 816 previously published sequences from contemporary Icelanders covering the same part of the control region [2],[8] revealed matches for only 23 (40.4%) of the 57 haplotypes from the skeletal remains. We note in this context that the contemporary Icelandic mtDNA pool is relatively well sampled. Thus, using the deCODE Genetics genealogical database to trace the matrilineal relationships of the 816 contemporary sequence donors to all other members of the population, we were able to determine that this sample represents about half of the sequences carried by all known Icelanders born after 1900 (189,086 of 374,960 individuals, descended from 541 common matrilineal ancestors).

The number of matches observed is significantly lower than would be expected under the assumption that the sequences from the IEMS represent a random sample of contemporary Icelandic sequences. To demonstrate this, a haplotype-match test was performed as follows. First, we randomly partitioned the 816 contemporary Icelandic sequences into one million samples of 68 and 748 sequences. The mean *k* for these samples of 68 sequences was 47.1 and the mean number of matches between the two sub-samples was 36.8. Next, we estimated the probability of obtaining a number of matches between the two sub-samples of contemporary sequences that was equal to or smaller than that observed between the IEMS and the samples of 748 contemporary sequences (a mean of 22.6 matches). This probability was 3.0×10^−6^, indicating that the IEMS came from an mtDNA pool different from that of contemporary Icelanders.

Given this difference, it is of interest to compare the IEMS with other contemporary European groups, in particular the original source populations from the British Isles and Scandinavia [1],[3]. Unfortunately, very few sequences spanning the sites 16055-16410 and 16517-334 are available for analysis from these populations. In order to balance the conflicting goals of maximising both the number and length of sequences, the region between sites 16055 and 16391 was used in these analyses, yielding a total of 10,503 sequences from various Eurasian populations that were obtained from the literature (see Table 2). For this shorter region, the 68 IEMS sequences were grouped into 48 mutationally distinct haplotypes (GD = 0.9754), and of these, 41 matched sequences from the contemporary population samples.

**Table 2:** Comparison of Icelandic early medieval sample to sequences from contemporary populations^a^.

			Comparison with the Icelandic early medieval sample			
REGION	N	k	Number of haplotype matches	Haplotype-match test P-value	Permutation Fisher Exact test P-value	AMOVA FST (P-value)
Iceland	947	172	26	**0.0032**	**<10−5**	−0.0022 (0.76)
North Atlantic Islands	927	270	26	**0.0088**	**0.016**	−0.0013 (0.678)
Mainland Scotland & Ireland	1215	402	31	0.379	0.494	−0.0033 (0.944)
Scandinavia	898	337	26	0.085	0.252	−0.0002 (0.38)
Northwest Europe	1374	536	30	0.388	0.498	0.0017 (0.175)
Northeast Europe	436	199	14	**<10−6**	**0.00072**	0.0076 **(0.014)**
European Russia	953	311	21	**0.000081**	**<10−5**	0.0111 **(0.00098)**
Southwest Europe	1825	736	31	0.639	0.882	0.0004 (0.343)
Southeast Europe	307	170	13	**0.000073**	0.099	−0.0017 (0.724)
Eastern Europe	869	395	27	0.403	0.34	0.0039 (0.059)
Near East	304	188	9	**<10−6**	**<10−5**	0.0121 **(<10−5)**
Northwest Africa	448	203	16	**0.000041**	**0.0004**	0.0032 **(0.045)**

a The references for the published sequences used for this table and a more detailed breakdown of the populations from which they derive can be found in Table S10.

As was seen for the longer sequence region, the number of matches (*m*) between the IEMS and the contemporary Icelandic sample (*m* = 26) is significantly smaller than expected if the IEMS were drawn from the same underlying mtDNA pool (*P* = 0.0032). A similar result is obtained when the IEMS is compared to the sample from the North Atlantic islands (which includes closely related populations from the Faroe Islands and the Western Isles) and in comparisons with five other more geographically distant groups. Interestingly, we find 26 or more haplotype matches between the IEMS and the source populations from Mainland Scotland and Ireland (*m* = 31) and Scandinavia (*m* = 26) and three other geographical regions within Europe (see Table 2). For these five groups, the null-hypothesis of the haplotype-match test cannot be rejected. Intriguingly, this indicates a closer relationship between the IEMS and the mtDNA pools of these five groups than between the IEMS and contemporary Icelanders. Similar results were obtained from applying the permutation version of the Fisher Exact test, which evaluates the null-hypothesis that two sets of sequences are random samples from the same population (Table 2).

The relationship between the IEMS and the contemporary groups was also assessed using the AMOVA statistic, which takes account of the number of mutational differences between sequences (Table 2). This revealed that, unlike the comparison with groups from four more geographically distant regions (Northeast Europe, European Russia, Northwest Africa and the Near East), the difference between the IEMS and contemporary Icelanders is not phylogenetically structured. Rather, the difference between these two samples (and between the IEMS and North Atlantic islands) is primarily one of haplotype frequencies. We note that this finding provides further support for the authenticity of the sequences assigned to the IEMS, as one would expect the primary source of contaminant DNA molecules to be from those who have handled the remains [17], i.e. contemporary Icelanders.

## Discussion

When interpreting the results obtained from the preceding population genetics analyses, it is important to bear in mind that mtDNA variation is weakly stratified among European populations [1]. For this reason, it is somewhat surprising to observe significant differences between the IEMS and any closely related contemporary populations. However, the population size of Iceland and the other North Atlantic islands has been small during the last 1100 years, compared to the other groups included in these analyses. Accordingly, there is evidence that the gene pools of these small populations have been more heavily affected by genetic drift than those of larger European populations [2],[9]. In the case of highly variable genetic systems, such as the mtDNA control region, this resulted in a faster rate of haplotype extinction and greater fluctuation in the frequencies of surviving haplotypes in the smaller populations [10]. This is the most straightforward explanation of the closer relationship between the IEMS and present-day samples from the source populations of Scotland, Ireland and Scandinavia (and three other regions of Europe) than to the descendants of the IEMS, contemporary Icelanders. Thus, because of faster drift induced evolution in Iceland, it appears that the haplotypes carried by the settlers have been better preserved in the source mtDNA pools of Scotland, Ireland and Scandinavia (and three other regions of Europe) than among contemporary Icelanders, the descendants of the settlers.

Ancient DNA studies have already demonstrated their potential to contribute to our understanding of macro-evolutionary history, particularly when it comes to elucidating the relationships between extant and extinct species [24]–[26]. Typically, the questions posed in such studies can be addressed using only a handful of samples, a situation that is well-suited to the labour intensive nature of this research. However, as shown in this report, ancient DNA can also be used to shed light on the rate of evolution and events that have occurred within populations over short periods of time. This requires relatively large sample sizes and is technically challenging, particularly in humans due to the problem of contamination. However, when combined with the inferential power of population genetics, ancient DNA data could be used to address a wide range of problems pertaining to the evolutionary history of past and present populations and the genealogical relationships between them.

## Materials and Methods

### Selection of Skeletal Remains for DNA Extraction

All the skeletal remains sampled in this study are stored at the National Museum of Iceland and were unearthed as a result of either archaeological excavations, soil erosion or construction work during the period 1880–1996. Although there is no formal record of the treatment of remains after excavation, it is clear that all the remains have been washed at some point and handled numerous times (proportional to the time from excavation) by a limited number of specialists. The National Museum's skeletal collection contains the remains of over 780 individuals, belonging to all periods of Icelandic history. Unfortunately, only a handful of these remains have been subjected to carbon dating. However, the archaeological context of each site can be effectively used to place most of the skeletal remains as belonging to individuals that died either before or after 1000 AD. This is because Icelanders converted to Christianity in the year 1000 AD, which entailed a major change in burial practices [27],[28]. Under the Norse religion, individuals were buried in peripheral sites belonging to their home farms, typically along with various artefacts that were considered potentially useful in the afterlife depending on sex and status (such as weapons, jewellery, animals, boats or household goods). Such graves typically exhibit a north-south orientation and are often found alone, but occasionally in clusters that may be interpreted as family plots. The move to Christianity saw the emergence of relatively large cemeteries shared by numerous farms and east-west orientated graves containing no grave goods [27],[28].

Although the classification of skeletal remains into pre- and post-1000 AD based on burial site context cannot be viewed as conclusive, there is nonetheless reason to believe that this scheme is reliable. Thus, of the handful of skeletal remains classified as pre-Christian (based on site context) that have been subjected to carbon-dating, all yield dates indicating death prior to 1000 AD [28]. Furthermore, a study of strontium isotope ratios has shown that of 46 supposed pre-Christian remains that were examined, 12 (26%) are from individuals that were born and bred outside Iceland – that is, likely first generation settlers [28].

Our objective was to sample teeth from as many pre-Christian remains as possible, to obtain a sizeable sample of mtDNA sequences from individuals that died soon after the settlement period (870–930 AD). Of the roughly 180 skeletal remains classed as pre-Christian at the National Museum, only 91 were available for sampling, or included teeth that could be sampled, or were sufficiently well preserved to be worth sampling. One tooth was sampled from 88 of these pre-Christian remains and two teeth were sampled from three of them (HSJ-A1, SSG-A1 and FOV-A1) in order to perform independent extractions for quality control.

In addition, one tooth each was sampled from four skeletal remains classed as Christian or unknown. Two of these remains (ÞSK-A1 and ÞSK-A26) were from the same cemetery at Skeljastaðir that is known to have been abandoned in the wake of a volcanic eruption of Mount Hekla in 1104. The remains from this cemetery therefore date from the first years of Christianity in Iceland, with one radiocarbon date from the late 10^th^ or early 11^th^ centuries [28]. Consequently, we elected to include the two remains from the Skeljastaðir cemetery in the pre-Christian sample for population analyses. The other two remains (TGS-A1 and SSS-A1) were recently discovered and of unknown temporal provenance, albeit most likely post 1000 AD.

In total, 98 teeth were sampled from 95 different skeletal remains that derive from 67 different sites with excavation dates spanning 1880–1996 (see Table S6).

### DNA Extraction in Reykjavik

Extraction of DNA was undertaken with strict procedures to minimize the potential for contamination, in a dedicated pre-PCR laboratory located at the storage premises of the National Museum of Iceland. No PCR has ever been performed in this building and neither has any other kind of molecular genetic research ever been undertaken there. Special care was taken never to visit the pre-PCR laboratory the same day after having been in an environment where PCR amplifications are performed or post-PCR work occurs. Sterile gloves, face masks, sterile reagents, pipette filter tips and frequent bleaching of the working surfaces and equipment were some of the precautions adopted during the extraction process.

DNA extractions were performed in Reykjavik for 97 of the 98 sampled teeth, representing all 95 skeletal remains included in the study. One of two teeth from the skeletal remain FOV-A1 was analysed only in the Barcelona laboratory. Extractions were carried out in batches of 8–10 teeth with one blank extraction in each batch to monitor the presence of contaminant DNA. Each tooth was powdered in an analytical mill. Half of the powder was retained to allow for a second DNA extraction. The other half of the powder was immediately decalcified overnight with shaking in 10 ml of 0.5 M EDTA pH 8.0 at 37°C. After centrifugation the supernatant was removed and the remaining samples were incubated overnight at 50°C in a digestion mix containing 8.5 ml of water, 1 ml 5% SDS, 0.5 ml 1 M Tris-HCl pH 8.0 and 50 µl of 1 mg/ml proteinase K. Next, the digests were extracted three times, first with phenol, second with phenol-chloroform and third with chloroform, and the aqueous phase was concentrated by dialysis centrifugation using Centricon-30 microconcentrators (Amicon) to a final volume of 100–200 µl. The resulting DNA extracts were subsequently stored in the pre-PCR laboratory at −20°C.

### PCR, Cloning and Sequencing in the Reykjavik Laboratories

All PCR reactions were set up and sealed at the pre-PCR laboratory. In each reaction, 1 µl of extracted template DNA was subjected to 40 cycles of amplification in 25 µl volume containing 1 unit of Taq polymerase (Ampli Taq Gold, Perkin Elmer, Palo Alto, CA), 10× reaction buffer, 2.5 mM MgCl_2_, 0.2 mM dNTPs, 12 mg/ml of BSA and 20 pmoles of each pair of primer. After a one-time preliminary 12 min activation of the enzyme at 95°C, each cycle consisted of 1 min steps, with denaturation at 95°C, annealing from 52 to 55.5°C (depending on the primer pair used, see Tables S7 and S8) and extension at 72°C. Negative controls (extraction blanks and PCR blanks) were subjected to amplification along with the ancient DNA extracts, to monitor contamination. No positive controls were used.

Four primers were combined in three different pairs to amplify the mtDNA control region between sites 16055 and 16410 (hypervariable segment 1, HVS1) in two short fragments (16055-16218 and 16209-16410) and one long fragment (16055-16410) that perfectly overlaps the combined shorter fragments. Five primers were combined in five different pairs to amplify the mtDNA control region between sites 16517 and 334 or 409, in two short fragments (16517-160 and 183-334/409) and one long fragment (16517-334/409) that intersects the combined shorter fragments between sites 16517 and 334. In this study the primers H334 and H409 were used interchangeably, as very few polymorphisms are known between sites 334 and 409 in humans.

PCR products were subjected to electrophoresis in 0.8% low-melting agarose gels in TA buffer and visualized with ethidium bromide staining. Positive amplification bands were excised from the gels, melted at 65°C for 20 min and eluted in 100–200 µl of sterile water, depending on the intensity of the band. The eluted gel-cuts were purified with MinElute Gel Extraction Kit (Qiagen Inc) and cloned into TOPO TA 2.1 vector system (Invitrogen Inc.). Plasmids were purified with Montage Plasmid Miniprep96 Kit (Millipore Inc.) and DNA sequenced with BigDye chemistry and analyzed on ABI3730 genetic analyzer (Applied Biosystems Inc.) using the same primers as in the PCR amplification.

### Replication in Barcelona: DNA Extraction, PCR, Cloning, and Sequencing

Twelve tooth samples were sent to the Barcelona lab for independent replication. Eleven of these samples (VDP-A6, BRE-A1, FOV-A1, GRV-A1, HBS-A6, KHF-A1, NNM-A1, SHS-A1, SUB-A1, STK-A1 and UAM-B1) were in the form of powder from half a tooth, retained during the initial step of DNA extraction in the Reykjavik laboratory. The twelfth sample was a whole tooth sampled from the skeletal remains FOV-A1. Extractions were performed in a dedicated ancient DNA laboratory with positive air pressure and nightly UV-irradiation, which is located in a building that is otherwise free from other molecular work. Samples were first powdered by grinding; 10 ml EDTA (pH:8; 0.5 M) were added to the powder overnight at 37°C to remove mineral salts; after centrifugation, the EDTA was poured off and the powder was incubated overnight at 50°C in a lysis solution (1 ml SDS 5%, 0.5 ml TRIS 1 M, 8.5 ml H_2_O and 1 mg/ml of proteinase K). Then the samples were extracted three times with phenol, phenol-chloroform and chloroform-isoamilic alcohol and concentrated with centricons (Millipore, 30,000 Daltons molecular cut-off) up to a 50–100 µl volume. Mock samples were included along the process to control for contamination.

Several different fragments of the mtDNA control region were amplified in 25 µl reactions with 1 µl to 5 µl of extract (some extracts were subjected to 1∶3 dilution in order to overcome inhibitors), 2 U AmpliTaq Gold (ABI, USA), 1× AmpliTaq Gold buffer (ABI, USA), 2.5 mM MgCl_2_ (ABI, USA), 1.4 mg/ml BSA, 0.2 mM dNTṔs and 1 µM of each primer (see Tables S7 and S8). The PCR reactions were subjected to 40 amplification cycles consisting of three steps (1 minute at 94°C, 1 minute at 50°C and 1 minute at 72°C) with an initial denaturing step at 94°C for 2 min and a final elongating step for 7 min at 72°C. One blank (e.g. sample with no template DNA) was included in the reaction for each ancient sample to monitor for possible contamination in the reagents. PCR products were electrophoresed in 1% low-melting point agarose gels (Invitrogen), stained with ethidium bromide. Amplification bands with the expected size were excised from the gel, purified with a silica extraction (Biotools) and cloned using the Topo TA cloning kit (Invitrogen) following the manufacturer's instructions. White colonies were harvested and subjected to PCR with M13 universal primers; inserts with the right size were subsequently sequenced with an Applied BioSystems 3100 DNA sequencer.

### Sequence Verification and Assignment to Samples

A key task in ancient DNA research is to determine the nucleotide sequence of the endogenous template molecules from a sampled tissue. Typically, researchers are faced with a set of different sequences covering a particular genomic fragment, obtained from cloned PCR products or some other method that yields sequences which reflect individual template molecules that were endogenous to the original sample or from an external source of contamination. Some templates may have been altered by nucleotide damage, resulting in apparently novel mutations on the background of the original sequence of the template molecules. The most common form of nucleotide damage is cytosine deamination [29],[30], resulting in sequences carrying additional C→T mutations in PCR-based approaches when the damaged nucleotides occurred on the template strand that is complementary to the sequencing primer (and G→A transitions observed when they occurred on the reverse strand). Other sequences may represent novel combinations of template molecules (endogenous or exogenous, damaged or undamaged), artificially fused together as a result of so-called “jumping PCR” events that may take place during amplification reactions. The configuration of sequences encountered for particular fragment of the genome from a single sample is a function several unknown variables. Among the most important of these is the concentration and condition of endogenous template molecules in the DNA extract, and the degree to which it has been contaminated with exogenous DNA template molecules. Accordingly, the task of identifying the sequence of the true endogenous template molecules is not straightforward – as any sequence encountered could be derived from an exogenous contaminant template molecule or could be from a damaged version of the endogenous template.

We used the following strategy to determine the sequence of samples spanning the mtDNA regions 16055-16410 and 16517-334, based on sequenced clones from the six aforementioned fragments. First, we used a recently published statistical test [31] to determine whether the pattern of mutational differences among a set of clone sequences, obtained from a particular fragment for a single sample, are consistent the presence of lesion-induced substitutions caused by cytosine deamination events. As the accumulation of cytosine deamination events is a function of time and the rate of exposure to hydrolytic damage, it is expected that clone sequences derived from ancient DNA templates will carry more lesion-induced substitutions than those derived from contaminant templates. Although there may be rare instances of greater lesions on contaminant templates [17], in most cases the supposed endogenous DNA molecules carry the strongest signal of cytosine deamination lesions [31]. The test is based on the *c*-statistic, which is calculated for each observed sequence (and intermediate sequences reconstructed using the median-joining algorithm [32]) and reflects the number of cytosine deamination events that are predicted to have occurred on the background of the sequence, under the assumption that the other clone sequences are damaged versions of it [31]. A permutation procedure is used to evaluate the statistical significance of *c* values for each sequence, and for the maximum value across all sequences, against the null-hypothesis that the pattern of mutations among sequences is random. A useful corollary of this approach is that the sequences with the greatest *c* values are natural candidates for being the undamaged version of the endogenous template DNA (particularly those with statistically significant *c* values). The *c*-statistic was therefore applied to the clone sequences obtained for each fragment from each sample with the aim of identifying an initial set of candidates for the endogenous sequences.

These candidate sequences were then evaluated in two different ways. First, it was determined whether candidate sequences from overlapping fragments (i.e. 16055-16218 or 16209-16410 vs. 16055-16410 and 16517-160 or 183-334 vs. 16517-334) in the same sample were identical. When true, this was taken as support for the authenticity of the candidate sequence for the shorter of the two overlapping fragments. When false, it was concluded that one (or both) of the candidate sequences was incorrect and further examination was needed. The same kind of analysis was also performed with relaxed matching conditions, where mutational differences between overlapping candidate sequences were ignored when likely to be due to cytosine deamination events (i.e. C↔T or G↔A differences), rare PCR misincorporation events or when they were insertions or deletions at the highly unstable sites 16183 and 302. This was done only when the overlapping candidate sequences shared key diagnostic mutations and when the suspected surplus mutations were at unusual sites or produced a phylogenetically unusual combination of known mutations.

The reasoning underlying this procedure is best described with an example. Thus, overlapping candidate sequences from sample STK-A2 for fragments 16209-16410 (motif: 16223T 16391A) and 16055-16410 (motif: 16072T 16129A 16223T 16254G 16278T 16287T 16290T 16391A) match for the mutations 16223T 16391A, a well known combination which is diagnostic of membership in haplogroup I. However, nothing close to the combination of mutations in the 16055-16410 candidate sequence is known from the literature. The three mutations 16278T 16287T 16290T bear the hallmark of being due to cytosine deamination events, while the difference at position 16254 may represent a misincorporation event. We note that only three clone sequences were retrieved for the 16055-16410 fragment and c_MAX_ was not statistically significant. The relaxing of matching conditions was more often carried out for candidate sequences from the longer fragments (16055-16410 and 16517-334/409), which tended to yield fewer clone sequences, reflecting a small number of underlying template molecules and resulting in lower power for c-statistic approach to identify the original sequence state of the endogenous template molecules.

Second, we implemented a procedure to test the phylogenetic compatibility of the candidate sequences proposed for non-overlapping fragments from the same samples. In short, we applied a rule-based approach to classify the candidate sequences obtained for each sample into the following haplogroups: H, I, J, K, T, U5, U3, U2, U4, V, W, X, Z and C (see Table S9 for the lists of rules for the six different fragments). In this procedure, each sequence is initially assigned a score vector for the assayed haplogroups, with each element set to 0, and then evaluated in relation to a set of haplogroup assignment rules. Each rule represents a test for the presence or absence of one or more alleles that are informative about haplogroup status, and carries with it a vector of scores that is added to the sequence score vector if evaluated as true. After all rules have been evaluated, the sequence score vector is examined, and haplogroups with scores less than the maximum are excluded. When this procedure is applied to candidate sequences from multiple fragments obtained from a sample, a consensus haplogroup assignment can be made for the sample based on the haplogroup(s) that were most often assigned across all fragments and incompatible fragment pairs can be identified for further examination.

The next step was a qualitative assessment of the sequences for each fragment from the remaining samples. For samples with incompatible candidate sequences, an attempt was made to select alternative candidate sequences from the clone sequences (or reconstructed median-vector sequences) available for one or more of the incompatible fragments. When incompatibility was observed between overlapping fragments (for example, 16055-16218 and 16055-16410) and a candidate sequence was available for the other short fragment (i.e. 16209-16410), then this extra information was utilized. Thus, if the longer fragment was incompatible with both shorter fragments, it was assumed that the candidate sequence for the longer fragment was erroneous. Conversely, if the longer fragment was compatible with the other short fragment, then the candidate sequence for the first shorter fragment was considered erroneous. When incompatibility was observed between the haplogroup designations of candidate sequences for different fragments, the majority-based consensus haplogroup designation for the sample was taken to be correct and alternative candidate sequences were sought for the fragments that conflicted with this designation. Samples with no apparent incompatibilities were also checked – in particular those that did not yield clone sequences for all six fragments and therefore had fewer than four overlapping fragments.

In general, a minimum number of changes were sought to achieve compatibility and when selecting fragments for change, more weight was given to candidate sequences from shorter fragments, to those with the greatest number of clone sequences and those with the most significant c_MAX_ value. When selecting a new candidate sequence for a particular fragment, preference was given to sequences with relatively high values of the c-statistic and those with the minimal number of cytosine deamination-like mutations at unusual sites. If no compatible candidate sequence could be selected from the clone sequences available for an incompatible fragment, the entire fragment was disregarded in further analyses.

A final method of evaluation, replication, was applied to a subset of the samples to determine whether candidate sequences selected on the basis of the preceding procedures were present among clone sequences from more than one amplification of the same fragment from either independent DNA extractions in different laboratories, independent DNA extractions in the same laboratory or different amplifications from the same extract. The presence of a candidate sequence in more than one amplification was viewed as a confirmation of sequence assignment to the sample in question.

## Supporting Information

Table S1 List of sampled skeletal remains and overview of experiments performed in the Reykjavik lab.
(0.44 MB DOC)

Table S2 All complete clone sequences obtained from PCRs in Reykjavik (R) and Barcelona (B).
(0.51 MB XLS)

Table S3 Initial c_MAX_ candidate sequences and changes made as a result of the qualitative assessment procedure.
(0.16 MB DOC)

Table S4 Results from replication amplifications performed in Reykjavik.
(0.08 MB DOC)

Table S5 Results from replication amplifications performed in Barcelona.
(0.05 MB DOC)

Table S6 List of sampled skeletal remains with background information.
(0.19 MB DOC)

Table S7 Oligonucleotide primer sequences.
(0.04 MB DOC)

Table S8 Primer pairs used for DNA amplifications.
(0.04 MB DOC)

Table S9 Rules for haplogroup classification of sequences.
(0.30 MB DOC)

Table S10 Published mtDNA sequences used in comparative analyses (sites 16055-16391).
(0.13 MB DOC)
