# Supplementary material for: Sequences From First Settlers Reveal Rapid Evolution in Icelandic mtDNA Pool
Source: PLoS Genet. 2009 Jan 16;5(1):e1000343. doi: 10.1371/journal.pgen.1000343 (PMC2613751; doi:10.1371/journal.pgen.1000343)
Supplement: Table S1 — List of sampled skeletal remains and overview of experiments performed in the Reykjavik lab. (0.44 MB DOC) [file pgen.1000343.s001.doc]

Table S1. List of sampled skeletal remains and overview of experiments performed in the Reykjavik lab

|  | **16055-16218** | | | | **16055-16410** | | | | **16209-16410** | | | | **16517-160** | | | | **16517-334/409** | | | | **183-334/409** | | | | **Total** | | | |
| --- | --- | --- | --- | --- | --- | --- | --- | --- | --- | --- | --- | --- | --- | --- | --- | --- | --- | --- | --- | --- | --- | --- | --- | --- | --- | --- | --- | --- |
| **Skeletal remain (tooth number)** | Attempted PCRs | Successful PCRs | Cloned PCRs | Total sequences | Attempted PCRs | Successful PCRs | Cloned PCRs | Total sequences | Attempted PCRs | Successful PCRs | Cloned PCRs | Total sequences | Attempted PCRs | Successful PCRs | Cloned PCRs | Total sequences | Attempted PCRs | Successful PCRs | Cloned PCRs | Total sequences | Attempted PCRs | Successful PCRs | Cloned PCRs | Total sequences | Attempted PCRs | Successful PCRs | Cloned PCRs | Total sequences |
| ABH-A1 | 4 | 1 | 1 | 25 | 3 | 1 | 0 | 0 | 3 | 3 | 1 | 0 | 2 | 2 | 1 | 13 | 3 | 3 | 0 | 0 | 4 | 3 | 0 | 0 | 19 | 13 | 3 | 38 |
| AEY-A1 | 3 | 1 | 1 | 0 | 2 | 1 | 0 | 0 | 2 | 1 | 1 | 12 | 2 | 2 | 1 | 1 | 3 | 1 | 0 | 0 | 5 | 2 | 1 | 0 | 17 | 8 | 4 | 13 |
| ASS-A1 | 3 | 3 | 1 | 4 | 3 | 2 | 2 | 12 | 3 | 3 | 1 | 12 | 2 | 2 | 2 | 12 | 3 | 3 | 1 | 1 | 4 | 3 | 1 | 12 | 18 | 16 | 8 | 53 |
| BAJ-A1 | 3 | 2 | 1 | 13 | 3 | 1 | 1 | 10 | 3 | 3 | 1 | 5 | 2 | 2 | 1 | 9 | 3 | 3 | 1 | 6 | 2 | 1 | 0 | 0 | 16 | 12 | 5 | 43 |
| BBE-A1 | 4 | 2 | 1 | 10 | 3 | 1 | 1 | 11 | 3 | 2 | 1 | 0 | 2 | 2 | 1 | 10 | 3 | 1 | 1 | 8 | 2 | 2 | 0 | 0 | 17 | 10 | 5 | 39 |
| BRE-A1 | 4 | 2 | 1 | 24 | 3 | 3 | 2 | 10 | 3 | 3 | 2 | 8 | 2 | 2 | 1 | 12 | 3 | 3 | 1 | 6 | 4 | 2 | 1 | 0 | 19 | 15 | 8 | 60 |
| BRV-A3 | 5 | 1 | 1 | 12 | 4 | 0 | 0 | 0 | 3 | 1 | 1 | 13 | 4 | 4 | 1 | 5 | 3 | 1 | 0 | 0 | 4 | 1 | 1 | 0 | 23 | 8 | 4 | 30 |
| BSE-A1 | 4 | 2 | 1 | 6 | 2 | 2 | 2 | 13 | 3 | 2 | 1 | 15 | 2 | 2 | 1 | 8 | 3 | 3 | 0 | 0 | 4 | 2 | 1 | 10 | 18 | 13 | 6 | 52 |
| DAP-A1 | 4 | 3 | 1 | 25 | 2 | 2 | 2 | 11 | 3 | 3 | 2 | 10 | 2 | 2 | 2 | 12 | 3 | 2 | 0 | 0 | 5 | 4 | 1 | 11 | 19 | 16 | 8 | 69 |
| DAV-A1 | 4 | 3 | 1 | 12 | 3 | 2 | 1 | 8 | 3 | 3 | 1 | 0 | 2 | 2 | 1 | 9 | 3 | 2 | 0 | 0 | 4 | 2 | 1 | 8 | 19 | 14 | 5 | 37 |
| DAV-A2 | 4 | 3 | 1 | 0 | 2 | 2 | 1 | 0 | 3 | 3 | 1 | 0 | 2 | 2 | 1 | 11 | 3 | 1 | 1 | 6 | 4 | 3 | 1 | 8 | 18 | 14 | 6 | 25 |
| DAV-A4 | 3 | 3 | 1 | 11 | 5 | 2 | 1 | 7 | 3 | 3 | 1 | 0 | 2 | 2 | 1 | 2 | 3 | 1 | 0 | 0 | 4 | 3 | 1 | 2 | 20 | 14 | 5 | 22 |
| DAV-A6 | 1 | 1 | 1 | 1 | 3 | 1 | 0 | 0 | 2 | 2 | 1 | 2 | 2 | 1 | 1 | 12 | 2 | 0 | 0 | 0 | 3 | 0 | 0 | 0 | 13 | 5 | 3 | 15 |
| DAV-A7 | 1 | 1 | 1 | 10 | 3 | 1 | 1 | 0 | 2 | 2 | 1 | 22 | 2 | 2 | 1 | 11 | 1 | 1 | 1 | 6 | 4 | 3 | 1 | 8 | 13 | 10 | 6 | 57 |
| DAV-A8 | 2 | 2 | 1 | 13 | 2 | 2 | 1 | 12 | 2 | 2 | 2 | 42 | 3 | 2 | 2 | 35 | 1 | 1 | 1 | 0 | 4 | 3 | 1 | 12 | 14 | 12 | 8 | 114 |
| DAV-A9 | 2 | 2 | 1 | 12 | 2 | 2 | 1 | 10 | 2 | 2 | 1 | 8 | 2 | 2 | 1 | 10 | 2 | 0 | 0 | 0 | 4 | 4 | 1 | 12 | 14 | 12 | 5 | 52 |
| DKS-A1 | 2 | 2 | 1 | 9 | 2 | 2 | 1 | 10 | 1 | 1 | 1 | 8 | 2 | 2 | 1 | 12 | 1 | 1 | 1 | 3 | 4 | 4 | 1 | 12 | 12 | 12 | 6 | 54 |
| EFS-A1 | 2 | 2 | 1 | 12 | 3 | 2 | 1 | 0 | 2 | 2 | 1 | 41 | 2 | 2 | 2 | 22 | 2 | 0 | 0 | 0 | 4 | 2 | 1 | 12 | 15 | 10 | 6 | 87 |
| EIM-A1 | 1 | 1 | 1 | 13 | 2 | 1 | 1 | 0 | 1 | 1 | 1 | 13 | 2 | 2 | 1 | 10 | 2 | 0 | 0 | 0 | 4 | 3 | 1 | 10 | 12 | 8 | 5 | 46 |
| ENV-A1 | 1 | 1 | 1 | 6 | 1 | 1 | 1 | 0 | 2 | 2 | 1 | 11 | 1 | 1 | 1 | 11 | 2 | 0 | 0 | 0 | 4 | 4 | 1 | 9 | 11 | 9 | 5 | 37 |
| EVS-A1 | 2 | 2 | 1 | 11 | 1 | 1 | 1 | 11 | 2 | 1 | 1 | 43 | 2 | 1 | 1 | 12 | 2 | 2 | 1 | 4 | 4 | 3 | 1 | 12 | 13 | 10 | 6 | 93 |
| FOV-A1 | 1 | 1 | 1 | 9 | 2 | 1 | 1 | 6 | 2 | 2 | 2 | 12 | 2 | 1 | 1 | 13 | 1 | 1 | 1 | 4 | 4 | 2 | 2 | 11 | 12 | 8 | 8 | 55 |
| FSS-A1 | 1 | 1 | 1 | 11 | 2 | 2 | 1 | 11 | 1 | 1 | 1 | 12 | 1 | 1 | 1 | 12 | 2 | 2 | 1 | 4 | 4 | 3 | 1 | 12 | 11 | 10 | 6 | 62 |
| GGH-A1 | 2 | 2 | 1 | 13 | 2 | 2 | 1 | 10 | 1 | 1 | 1 | 12 | 1 | 1 | 1 | 9 | 2 | 0 | 0 | 0 | 4 | 3 | 1 | 11 | 12 | 9 | 5 | 55 |
| GRF-A1 | 2 | 2 | 1 | 6 | 3 | 3 | 1 | 7 | 2 | 2 | 1 | 8 | 1 | 1 | 1 | 11 | 4 | 2 | 1 | 10 | 1 | 1 | 1 | 9 | 13 | 11 | 6 | 51 |
| GRM-A1 | 2 | 2 | 1 | 12 | 2 | 1 | 1 | 57 | 1 | 1 | 1 | 35 | 2 | 2 | 2 | 53 | 2 | 2 | 2 | 21 | 3 | 2 | 2 | 76 | 12 | 10 | 9 | 254 |
| GRS-A1 | 2 | 2 | 1 | 12 | 2 | 1 | 1 | 12 | 2 | 2 | 1 | 12 | 1 | 1 | 1 | 10 | 1 | 1 | 1 | 9 | 4 | 4 | 1 | 0 | 12 | 11 | 6 | 55 |
| GRS-A2 | 2 | 2 | 1 | 13 | 2 | 2 | 1 | 22 | 2 | 2 | 1 | 12 | 2 | 2 | 1 | 10 | 1 | 1 | 1 | 0 | 3 | 2 | 1 | 11 | 12 | 11 | 6 | 68 |
| GRV-A1 | 1 | 1 | 1 | 13 | 2 | 1 | 1 | 0 | 1 | 1 | 1 | 10 | 2 | 2 | 1 | 10 | 2 | 2 | 1 | 10 | 4 | 3 | 1 | 10 | 12 | 10 | 6 | 53 |
| GTE-A1 | 1 | 1 | 1 | 12 | 3 | 1 | 1 | 0 | 2 | 2 | 1 | 10 | 2 | 1 | 1 | 7 | 4 | 3 | 2 | 3 | 1 | 0 | 0 | 0 | 13 | 8 | 6 | 32 |
| GTE-A2 | 2 | 2 | 1 | 6 | 3 | 2 | 1 | 13 | 2 | 2 | 1 | 10 | 2 | 1 | 1 | 12 | 4 | 3 | 1 | 0 | 1 | 0 | 0 | 0 | 14 | 10 | 5 | 41 |
| HBS-A6 | 2 | 2 | 1 | 2 | 3 | 2 | 1 | 2 | 2 | 2 | 1 | 11 | 2 | 2 | 1 | 8 | 5 | 4 | 1 | 12 | 1 | 0 | 0 | 0 | 15 | 12 | 5 | 35 |
| HRK-A1 | 2 | 2 | 1 | 4 | 4 | 0 | 0 | 0 | 2 | 1 | 1 | 7 | 3 | 0 | 0 | 0 | 5 | 0 | 0 | 0 | 2 | 0 | 0 | 0 | 18 | 3 | 2 | 11 |
| HRK-A2 | 2 | 2 | 1 | 13 | 3 | 1 | 1 | 11 | 2 | 2 | 1 | 10 | 2 | 1 | 1 | 7 | 5 | 0 | 0 | 0 | 1 | 1 | 1 | 17 | 15 | 7 | 5 | 58 |
| HSJ-A1 (1) | 6 | 3 | 1 | 0 | 4 | 2 | 1 | 13 | 3 | 3 | 1 | 0 | 2 | 2 | 1 | 0 | 2 | 2 | 1 | 0 | 4 | 3 | 1 | 12 | 21 | 15 | 6 | 25 |
| HSJ-A1 (2) | 2 | 2 | 2 | 20 | 3 | 3 | 1 | 0 | 2 | 2 | 1 | 12 | 2 | 1 | 1 | 42 | 5 | 3 | 2 | 38 | 1 | 0 | 0 | 0 | 15 | 11 | 7 | 112 |
| HVL-A2 | 2 | 2 | 1 | 12 | 3 | 1 | 1 | 0 | 1 | 1 | 1 | 0 | 1 | 1 | 1 | 18 | 4 | 1 | 1 | 0 | 2 | 1 | 0 | 0 | 13 | 7 | 5 | 30 |
| KHF-A1 | 2 | 2 | 1 | 3 | 3 | 2 | 2 | 0 | 1 | 1 | 1 | 4 | 2 | 1 | 1 | 4 | 4 | 1 | 0 | 0 | 3 | 3 | 1 | 11 | 15 | 10 | 6 | 22 |
| KNS-A1 | 2 | 2 | 1 | 13 | 3 | 2 | 1 | 14 | 2 | 2 | 1 | 6 | 2 | 2 | 1 | 13 | 4 | 1 | 1 | 3 | 1 | 1 | 1 | 15 | 14 | 10 | 6 | 64 |
| KRE-A1 | 2 | 2 | 1 | 11 | 2 | 2 | 1 | 10 | 2 | 2 | 1 | 11 | 1 | 1 | 1 | 12 | 4 | 4 | 1 | 2 | 1 | 1 | 1 | 5 | 12 | 12 | 6 | 51 |
| KVE-A1 | 2 | 2 | 1 | 10 | 3 | 3 | 2 | 4 | 2 | 1 | 1 | 8 | 2 | 2 | 1 | 12 | 4 | 1 | 1 | 6 | 1 | 1 | 1 | 5 | 14 | 10 | 7 | 45 |
| LKH-A1 | 2 | 2 | 1 | 8 | 3 | 2 | 1 | 9 | 2 | 2 | 1 | 10 | 2 | 1 | 1 | 7 | 4 | 3 | 1 | 0 | 1 | 1 | 1 | 11 | 14 | 11 | 6 | 45 |
| MKL-A1 | 2 | 2 | 1 | 13 | 2 | 1 | 0 | 0 | 2 | 2 | 2 | 42 | 2 | 2 | 1 | 7 | 4 | 1 | 1 | 33 | 1 | 1 | 1 | 12 | 13 | 9 | 6 | 107 |
| MKR-A1 | 2 | 2 | 1 | 13 | 3 | 1 | 1 | 10 | 2 | 2 | 1 | 11 | 2 | 1 | 1 | 11 | 4 | 2 | 1 | 4 | 1 | 1 | 1 | 12 | 14 | 9 | 6 | 61 |
| NNM-A1 | 2 | 2 | 1 | 10 | 4 | 1 | 0 | 0 | 2 | 2 | 1 | 11 | 2 | 1 | 1 | 4 | 4 | 1 | 1 | 12 | 1 | 1 | 1 | 12 | 15 | 8 | 5 | 49 |
| NÞR-A1 | 2 | 2 | 1 | 12 | 3 | 2 | 1 | 0 | 2 | 2 | 2 | 27 | 2 | 2 | 2 | 26 | 5 | 0 | 0 | 0 | 3 | 2 | 2 | 20 | 17 | 10 | 8 | 85 |
| NÞR-A2 | 2 | 2 | 1 | 11 | 2 | 1 | 1 | 2 | 2 | 2 | 2 | 24 | 3 | 1 | 1 | 12 | 5 | 1 | 1 | 3 | 1 | 1 | 1 | 12 | 15 | 8 | 7 | 64 |
| NUA-A1 | 2 | 2 | 1 | 13 | 3 | 2 | 1 | 1 | 2 | 2 | 2 | 24 | 2 | 2 | 2 | 20 | 5 | 0 | 0 | 0 | 1 | 1 | 1 | 11 | 15 | 9 | 7 | 69 |
| ORE-A1 | 2 | 2 | 1 | 12 | 3 | 3 | 1 | 12 | 1 | 1 | 1 | 9 | 1 | 1 | 1 | 7 | 4 | 3 | 0 | 0 | 1 | 1 | 1 | 12 | 12 | 11 | 5 | 52 |
| OXH-A2 | 2 | 2 | 1 | 12 | 3 | 3 | 1 | 0 | 2 | 2 | 1 | 11 | 1 | 1 | 1 | 3 | 4 | 1 | 0 | 0 | 1 | 1 | 1 | 4 | 13 | 10 | 5 | 30 |
| SAE-A1 | 2 | 2 | 1 | 7 | 2 | 1 | 1 | 1 | 2 | 2 | 1 | 12 | 2 | 1 | 1 | 11 | 4 | 2 | 1 | 11 | 1 | 1 | 1 | 12 | 13 | 9 | 6 | 54 |
| SBT-A1 | 2 | 2 | 1 | 13 | 3 | 3 | 1 | 11 | 2 | 2 | 1 | 9 | 2 | 2 | 1 | 12 | 3 | 3 | 0 | 0 | 1 | 1 | 1 | 11 | 13 | 13 | 5 | 56 |
| SFA-A1 | 2 | 2 | 1 | 13 | 3 | 1 | 1 | 10 | 2 | 2 | 1 | 12 | 2 | 1 | 1 | 12 | 3 | 1 | 1 | 2 | 1 | 1 | 1 | 5 | 13 | 8 | 6 | 54 |
| SFA-B1 | 2 | 2 | 1 | 11 | 2 | 2 | 1 | 1 | 2 | 1 | 1 | 13 | 2 | 2 | 1 | 12 | 4 | 2 | 1 | 11 | 1 | 1 | 1 | 14 | 13 | 10 | 6 | 62 |
| SFS-A1 | 2 | 2 | 1 | 12 | 3 | 2 | 1 | 10 | 2 | 2 | 1 | 10 | 2 | 2 | 1 | 10 | 3 | 1 | 1 | 0 | 1 | 1 | 1 | 11 | 13 | 10 | 6 | 53 |
| SHS-A1 | 2 | 2 | 1 | 8 | 3 | 2 | 1 | 4 | 2 | 2 | 1 | 8 | 2 | 2 | 1 | 8 | 3 | 1 | 1 | 7 | 3 | 3 | 3 | 19 | 15 | 12 | 8 | 54 |
| SSG-A1 (1) | 6 | 3 | 1 | 0 | 3 | 3 | 1 | 12 | 3 | 3 | 1 | 0 | 3 | 3 | 2 | 0 | 3 | 2 | 1 | 0 | 4 | 4 | 1 | 0 | 22 | 18 | 7 | 12 |
| SSG-A1 (2) | 2 | 2 | 1 | 25 | 3 | 3 | 0 | 0 | 2 | 2 | 1 | 21 | 2 | 2 | 1 | 20 | 3 | 2 | 1 | 10 | 1 | 1 | 1 | 22 | 13 | 12 | 5 | 98 |
| SSG-A2 | 2 | 2 | 1 | 12 | 4 | 1 | 1 | 0 | 2 | 2 | 1 | 24 | 3 | 1 | 1 | 12 | 4 | 0 | 0 | 0 | 1 | 1 | 1 | 5 | 16 | 7 | 5 | 53 |
| SSG-A3 | 2 | 2 | 1 | 13 | 2 | 2 | 1 | 1 | 2 | 1 | 1 | 9 | 1 | 1 | 1 | 9 | 4 | 2 | 0 | 0 | 1 | 1 | 1 | 10 | 12 | 9 | 5 | 42 |
| SSG-A4 | 2 | 2 | 1 | 13 | 3 | 3 | 2 | 11 | 2 | 1 | 1 | 5 | 2 | 1 | 1 | 11 | 4 | 0 | 0 | 0 | 1 | 1 | 1 | 8 | 14 | 8 | 6 | 48 |
| SSJ-A2 | 1 | 1 | 1 | 10 | 1 | 1 | 1 | 2 | 2 | 1 | 1 | 11 | 2 | 1 | 1 | 10 | 2 | 1 | 0 | 0 | 2 | 1 | 1 | 10 | 10 | 6 | 5 | 43 |
| SSS-A1 | 1 | 1 | 1 | 13 | 2 | 2 | 1 | 13 | 2 | 2 | 1 | 5 | 3 | 3 | 1 | 12 | 2 | 2 | 1 | 3 | 4 | 3 | 1 | 0 | 14 | 13 | 6 | 46 |
| SSV-A1 | 1 | 1 | 1 | 12 | 2 | 1 | 1 | 12 | 2 | 1 | 1 | 12 | 2 | 1 | 1 | 12 | 2 | 0 | 0 | 0 | 2 | 2 | 2 | 12 | 11 | 6 | 6 | 60 |
| STB-A1 | 1 | 1 | 1 | 9 | 1 | 1 | 1 | 9 | 1 | 1 | 1 | 6 | 1 | 1 | 1 | 10 | 2 | 1 | 0 | 0 | 2 | 2 | 1 | 6 | 8 | 7 | 5 | 40 |
| STH-A1 | 1 | 1 | 1 | 11 | 1 | 1 | 1 | 10 | 1 | 1 | 1 | 12 | 2 | 1 | 1 | 8 | 2 | 1 | 1 | 2 | 1 | 0 | 0 | 0 | 8 | 5 | 5 | 43 |
| STH-A2 | 1 | 1 | 1 | 13 | 1 | 1 | 0 | 0 | 2 | 1 | 1 | 12 | 2 | 1 | 1 | 11 | 2 | 0 | 0 | 0 | 2 | 1 | 1 | 11 | 10 | 5 | 4 | 47 |
| STK-A1 | 1 | 1 | 1 | 10 | 1 | 1 | 1 | 4 | 1 | 1 | 1 | 11 | 1 | 1 | 1 | 11 | 2 | 2 | 1 | 3 | 1 | 0 | 0 | 0 | 7 | 6 | 5 | 39 |
| STK-A2 | 2 | 1 | 1 | 11 | 2 | 1 | 1 | 3 | 2 | 1 | 1 | 31 | 2 | 0 | 0 | 10 | 1 | 1 | 1 | 0 | 2 | 1 | 1 | 14 | 11 | 5 | 5 | 69 |
| STT-A1 | 1 | 1 | 1 | 11 | 1 | 1 | 0 | 0 | 2 | 1 | 1 | 12 | 1 | 1 | 1 | 12 | 2 | 1 | 0 | 0 | 2 | 1 | 1 | 0 | 9 | 6 | 4 | 35 |
| STT-A2 | 1 | 1 | 1 | 8 | 2 | 1 | 1 | 9 | 1 | 1 | 1 | 11 | 1 | 1 | 1 | 7 | 2 | 0 | 0 | 0 | 2 | 1 | 1 | 11 | 9 | 5 | 5 | 46 |
| STT-A4 | 2 | 1 | 1 | 13 | 2 | 1 | 0 | 0 | 2 | 1 | 1 | 12 | 2 | 1 | 1 | 12 | 2 | 0 | 0 | 0 | 2 | 1 | 1 | 11 | 12 | 5 | 4 | 48 |
| SUB-A1 | 1 | 1 | 1 | 9 | 2 | 1 | 1 | 12 | 1 | 1 | 1 | 11 | 1 | 1 | 1 | 11 | 2 | 1 | 0 | 0 | 2 | 2 | 1 | 5 | 9 | 7 | 5 | 48 |
| SUB-B1 | 1 | 1 | 1 | 9 | 2 | 1 | 1 | 0 | 1 | 1 | 1 | 12 | 2 | 1 | 1 | 10 | 1 | 1 | 0 | 0 | 1 | 1 | 1 | 7 | 8 | 6 | 5 | 38 |
| SVE-A1 | 2 | 1 | 1 | 10 | 2 | 1 | 1 | 7 | 2 | 1 | 1 | 11 | 2 | 1 | 1 | 12 | 1 | 1 | 0 | 0 | 2 | 1 | 1 | 10 | 11 | 6 | 5 | 50 |
| SVK-A1 | 2 | 1 | 1 | 19 | 2 | 1 | 1 | 12 | 2 | 1 | 1 | 15 | 2 | 1 | 1 | 12 | 2 | 1 | 0 | 0 | 2 | 1 | 1 | 12 | 12 | 6 | 5 | 70 |
| SYK-A1 | 2 | 2 | 2 | 13 | 2 | 1 | 1 | 2 | 1 | 1 | 1 | 6 | 1 | 1 | 1 | 5 | 1 | 1 | 0 | 0 | 1 | 1 | 1 | 7 | 8 | 7 | 6 | 33 |
| SYR-A1 | 2 | 1 | 1 | 12 | 2 | 1 | 1 | 0 | 2 | 1 | 1 | 10 | 2 | 1 | 1 | 12 | 2 | 0 | 0 | 0 | 2 | 1 | 1 | 11 | 12 | 5 | 5 | 45 |
| SYR-B1 | 1 | 1 | 1 | 11 | 1 | 1 | 1 | 1 | 2 | 1 | 1 | 11 | 2 | 1 | 1 | 11 | 2 | 0 | 0 | 0 | 2 | 1 | 1 | 11 | 10 | 5 | 5 | 45 |
| TGS-A1 | 1 | 1 | 1 | 10 | 2 | 2 | 1 | 12 | 2 | 2 | 1 | 9 | 2 | 1 | 1 | 12 | 2 | 2 | 1 | 2 | 4 | 2 | 1 | 10 | 13 | 10 | 6 | 55 |
| ÞLS-A1 | 1 | 1 | 1 | 9 | 2 | 1 | 1 | 2 | 2 | 1 | 0 | 11 | 2 | 1 | 1 | 11 | 1 | 1 | 0 | 0 | 2 | 1 | 1 | 12 | 10 | 6 | 4 | 45 |
| THS-A1 | 1 | 1 | 1 | 22 | 1 | 1 | 1 | 6 | 1 | 1 | 1 | 10 | 1 | 1 | 1 | 13 | 2 | 1 | 0 | 0 | 2 | 2 | 1 | 0 | 8 | 7 | 5 | 51 |
| ÞSK-A1 | 1 | 1 | 1 | 13 | 2 | 1 | 1 | 13 | 2 | 2 | 1 | 7 | 2 | 2 | 1 | 11 | 2 | 1 | 0 | 0 | 4 | 3 | 1 | 8 | 13 | 10 | 5 | 52 |
| ÞSK-A26 | 2 | 2 | 1 | 12 | 2 | 2 | 0 | 0 | 2 | 2 | 1 | 7 | 2 | 2 | 1 | 11 | 2 | 2 | 0 | 0 | 2 | 1 | 1 | 12 | 12 | 11 | 4 | 42 |
| TMY-A1 | 1 | 1 | 1 | 11 | 1 | 1 | 1 | 6 | 1 | 1 | 1 | 3 | 1 | 1 | 1 | 8 | 2 | 1 | 0 | 0 | 2 | 1 | 1 | 12 | 8 | 6 | 5 | 40 |
| TMY-A2 | 1 | 1 | 1 | 13 | 1 | 1 | 1 | 5 | 2 | 1 | 1 | 36 | 2 | 1 | 1 | 10 | 2 | 1 | 1 | 0 | 2 | 1 | 1 | 10 | 10 | 6 | 6 | 74 |
| UAM-B1 | 1 | 1 | 1 | 3 | 2 | 0 | 0 | 0 | 1 | 1 | 1 | 9 | 1 | 1 | 1 | 8 | 1 | 1 | 1 | 3 | 1 | 1 | 1 | 7 | 7 | 5 | 5 | 30 |
| VDP-A3 | 1 | 1 | 1 | 18 | 2 | 1 | 1 | 11 | 1 | 1 | 1 | 11 | 1 | 1 | 1 | 10 | 1 | 1 | 0 | 0 | 2 | 1 | 1 | 6 | 8 | 6 | 5 | 56 |
| VDP-A5 | 1 | 1 | 1 | 10 | 2 | 1 | 1 | 20 | 2 | 0 | 1 | 0 | 2 | 1 | 1 | 6 | 1 | 1 | 1 | 9 | 2 | 1 | 1 | 12 | 10 | 5 | 6 | 57 |
| VDP-A6 | 1 | 1 | 1 | 7 | 1 | 1 | 1 | 2 | 1 | 1 | 1 | 11 | 1 | 1 | 1 | 8 | 1 | 1 | 0 | 0 | 1 | 1 | 1 | 5 | 6 | 6 | 5 | 33 |
| VDP-A7 | 1 | 1 | 1 | 12 | 1 | 1 | 1 | 2 | 1 | 1 | 1 | 12 | 1 | 1 | 1 | 12 | 1 | 1 | 0 | 0 | 1 | 1 | 1 | 12 | 6 | 6 | 5 | 50 |
| VDS-A1 | 1 | 1 | 1 | 11 | 2 | 0 | 0 | 0 | 2 | 1 | 1 | 11 | 2 | 1 | 1 | 11 | 1 | 0 | 0 | 0 | 2 | 1 | 1 | 11 | 10 | 4 | 4 | 44 |
| VSL-A1 | 1 | 1 | 1 | 12 | 2 | 0 | 0 | 0 | 1 | 1 | 1 | 0 | 1 | 1 | 1 | 10 | 1 | 0 | 0 | 0 | 1 | 1 | 1 | 11 | 7 | 4 | 4 | 33 |
| YGS-A2 | 1 | 1 | 1 | 12 | 2 | 1 | 1 | 0 | 1 | 1 | 1 | 9 | 2 | 1 | 1 | 5 | 1 | 1 | 0 | 0 | 2 | 1 | 1 | 9 | 9 | 6 | 5 | 35 |
| YGS-B1 | 1 | 1 | 1 | 10 | 1 | 1 | 1 | 9 | 1 | 1 | 1 | 13 | 2 | 1 | 1 | 9 | 1 | 1 | 0 | 0 | 2 | 1 | 1 | 11 | 8 | 6 | 5 | 52 |
| YGS-B2 | 1 | 1 | 2 | 33 | 1 | 1 | 1 | 9 | 1 | 1 | 0 | 4 | 1 | 1 | 1 | 10 | 1 | 0 | 0 | 0 | 1 | 1 | 1 | 11 | 6 | 5 | 5 | 67 |
| YGS-B5 | 1 | 1 | 1 | 13 | 2 | 0 | 0 | 0 | 1 | 1 | 1 | 0 | 2 | 0 | 0 | 0 | 1 | 0 | 0 | 0 | 2 | 1 | 1 | 0 | 9 | 3 | 3 | 13 |
| **Total** | 186 | 156 | 100 | 1090 | 223 | 140 | 88 | 605 | 180 | 154 | 103 | 1133 | 178 | 135 | 102 | 1069 | 249 | 121 | 48 | 277 | 223 | 154 | 91 | 863 | 1239 | 860 | 532 | 5037 |
