# Supplementary material for: Sequences From First Settlers Reveal Rapid Evolution in Icelandic mtDNA Pool
Source: PLoS Genet. 2009 Jan 16;5(1):e1000343. doi: 10.1371/journal.pgen.1000343 (PMC2613751; doi:10.1371/journal.pgen.1000343)
Supplement: Table S3 — Initial cMAX candidate sequences and changes made as a result of the qualitative assessment procedure. (0.16 MB DOC) [file pgen.1000343.s003.doc]

Table S3. Initial c_MAX_ candidate sequences and changes made as a result of the qualitative assessment procedure

| **Sample** | **Fragment** | **cMAX candidate sequence** | **C-score**  **(P-value)** | **Alternative candidate sequence** | **C-score**  **(P-value)** | **Difference between sequences**  **(CDS = cytosine deamination substitution, PMS = PCR misincorporation substitution, Indel = I, JP = Jumping PCR)** |
| --- | --- | --- | --- | --- | --- | --- |
| ASS-A-1 | 183-409 | 263G 302_1C 315_1C 338T | 19 (0) | 263G 315_1C | 13.167 (0) | CDS(338T), I(302_1C) |
| ASS-A-1 | 16055-16410 | 16270T | 6 (0.0116) | CRS | 4 (0.0188) | CDS(16270T) |
| BAJ-A-1 | 16517-409 | 16519C 73G 189G 195C 204C 207A 263G | 3.5 (0.0428) | 16519C 73G 189G 195C 204C 207A 263G 315_1C | 2.667 (0.0804) | I(315_1C) |
| BAJ-A-1 | 16517-160 | 16519C 61T 73G | 14 (0) | 16519C 73G | 9 (0) | CDS(61T) |
| BBE-A-1 | 16517-409 | 16519C 263G 302_1C 302_2C 315_1C | 4 (0.048) | 16519C 263G 302_2C 302_3C 315_1C | 4 (0.048) | I(302_2C) |
| BRE-A-1 | 16209-16410 | 16355T | 8 (0.0004) | CRS | 5 (0.0048) | CDS(16355T) |
| BSE-A-1 | 16055-16218 | 16069T 16126C 16147T | 9 (0) | 16069T 16126C | 7 (0.0012) | CDS(16147T) |
| BSE-A-1 | 183-409 | 185A 228A 263G 295T 302_1C 315_1C | 9.5 (0.0002) | 185A 228A 263G 295T 315_1C | 8.167 (0.002) | I(302_1C) |
| BSE-A-1 | 16055-16410 | 16129A 16239T | 0 (1) |  |  | Fragment excluded |
| DAV-A-1 | 16055-16218 | 16209C | 4.5 (0.035) | 16162G 16209C | 2.5 (0.1394) | CDS(16162A) |
| DAV-A-4 | 16055-16410 | 16129A 16337T 16339T 16354T | 28 (0) | 16129A | 28 (0) | CDS(16337T 16339T 16354T) |
| DAV-A-7 | 16517-409 | 73G 165G 263G 302_2C 315_1C | 1 (0.3282) | 16519C 73G 150T 263G 315_1C | 0 (1) | Different motif |
| DAV-A-8 | 16055-16410 | 16154C 16192T 16239T 16256T 16270T 16399G | 1 (0.4992) | CRS | 0 (1) | CDS(16107T 16360T 16366T 16376T) |
| DAV-A-8 | 183-409 | 263G 302_1C 315_1C | 6.75 (0) | 263G 315_1C | 6.167 (0) | Different motif |
| DAV-A-9 | 16517-160 | 16519C 112T 122T | 16.5 (0) | 16519C | 14.333 (0) | CDS(112T 122T) |
| DKS-A-1 | 16517-409 | 73G 150T 263G 302_1C 309T 315_1C | 0 (1) | 73G 150T 263G 302_1C 315_1C | 0 (1) | CDS(309T) |
| EFS-A-1 | 16209-16410 | 16231C | 2.5 (0.038) | 16239T | 2 (0.5182) | Different motif |
| EIM-A-1 | 16209-16410 | 16400T | 21 (0) | CRS | 20 (0) | CDS(16400T) |
| EVS-A-1 | 16055-16410 | 16356C | 1 (0.8842) | 16362C | 0 (1) | CDS(16236T) |
| EVS-A-1 | 16517-409 | 16519C 73G 195C 263G 302_1C 315_1C | 4 (0.0586) |  |  | Fragment excluded |
| FOV-A-1 | 16517-409 | 16519C 152C 263G 315_1C | 4 (0.0632) |  |  | Fragment excluded |
| GGH-A-1 | 16055-16410 | 16069T 16126C 16145A 16172C 16192T 16222T 16261T | 0 (1) | 16293G | 0 (1) | Different motif |
| GRF-A-1 | 183-409 | 185A 228A 263G 295T 308T 315_1C | 9 (0) | 185A 228A 263G 295T 315_1C | 6.5 (0) | CDS(308T) |
| GRF-A-1 | 16517-334 | 36A 73G 185A 228A 263G 295T 315_1C | 5 (0.0116) | 73G 185A 228A 263G 295T 315_1C | 4.5 (0.053) | CDS(36A) |
| GRS-A-1 | 16209-16410 | 16224C 16311C 16355T 16360T | 17 (0) | 16224C 16311C | 14.167 (0) | CDS(16355T 16360T) |
| GRV-A-1 | 16517-409 | 16519C 152C 263G 315_1C | 1 (1) |  |  | Fragment excluded |
| GRV-A-1 | 16055-16218 | CRS | 15.333 (0) | 16129A | 3 (0.2576) | Different motif |
| GTE-A-1 | 16517-409 | 16519C 16527T 41T 73G 150T 263G 315_1C | 1 (0.5006) | 263G 315_1C | 0 (1) | CDS(29T 33T 96T 132T 198T) |
| GTE-A-1 | 16517-160 | 112T | 8 (0) | CRS | 6 (0.0054) | CDS(112T) |
| GTE-A-2 | 16209-16410 | CRS | 5 (0.1252) | 16224C 16311C 16362C | 0 (1) | CDS(16267T 16294T 16306T 16408T) |
| HBS-A-6 | 16209-16410 | 16223T 16352C 16391A | 1.5 (0.2828) | 16223T 16391A | 1 (0.8858) | PMS(16352C) |
| HRK-A-1 | 16209-16410 | 16311C | 11 (0) | 16224C 16311C | 9.5 (0) | CDS(16224T) |
| HRK-A-2 | 16055-16410 | CRS | 4 (0.038) | 16069T 16126C 16145A 16172C 16192T 16222T 16261T | 3.333 (0.0396) | Different motif |
| HRK-A-2 | 16517-160 | 46C 152C | 2 (0.1024) | 73G | 2 (0.268) | Different motif |
| HVL-A-2 | 16055-16218 | 16057T 16099T | 13 (0) | CRS | 10.5 (0) | CDS(16057T 16099T) |
| KRE-A-1 | 16517-160 | 37G 73G | 1 (1) | 73G | 0 (1) | PMS(37G) |
| KRE-A-1 | 183-409 | 263G 315_1C | 0 (1) | 228A 263G 295T 302_1C 315_1C | 0 (1) | Different motif |
| KRE-A-1 | 16055-16410 | 16069T 16126C 16285G | 1.5 (0.5162) | 16069T 16126C | 1 (1) | PMS(16285G) |
| KRE-A-1 | 16055-16218 | 16069T 16126C 16202G | 1 (0.513) | 16069T 16126C | 0 (1) | PMS(16202G) |
| LKH-A-1 | 16055-16410 | 16126C 16174T 16294T 16296T 16304C | 13 (0.0002) | 16126C 16294T 16296T 16304C | 10.5 (0) | CDS(16174T) |
| MKL-A-1 | 16517-334 | 16531G 72C 263G 302_1C 315_1C | 1.5 (0.274) |  | () | Fragment excluded |
| MKL-A-1 | 16517-160 | 72C | 1 (0.5546) | 16519C 73G 143A | 1 (0.4934) | CDS(16528T 16560T 11T 44T 141T), PMS(16531G) |
| NNM-A-1 | 16055-16218 | 16093C | 1 (1) | CRS | 0 (1) | Different motif |
| NNM-A-1 | 16517-160 | 72C | 1 (1) | CRS | 0 (1) | Different motif |
| NÞR-A-2 | 16517-409 | 73G 263G 302_1C 315_1C | 2 (0.4966) |  |  | Fragment excluded |
| NÞR-A-2 | 16055-16410 | 16126C 16186T 16188T 16232T 16257T 16294T 16296T | 0 (1) |  |  | Fragment excluded |
| NÞR-A-2 | 16055-16218 | 16099T | 8 (0) | CRS | 5 (0.0002) | CDS(16099T) |
| NUA-A-1 | 16055-16410 | 16189C 16189_1C | 0 (1) | 16189C | 0 (1) | I(16189_1C) |
| NUA-A-1 | 16517-160 | 16519C 16549T 86T 146C | 14 (0) | 16519C 146C | 13.667 (0) | CDS(16549T 86T) |
| NUA-A-1 | 16055-16218 | 16184- 16189C | 6 (0) | 16189C | 5.5 (0.0088) | I(16184-) |
| NUA-A-1 | 16209-16410 | 16224C 16311C | 5.833 (0.0016) | CRS | 2 (0.6662) | Different motif |
| ORE-A-1 | 16055-16410 | 16239T | 1 (1) |  |  | Fragment excluded |
| OXH-A-2 | 183-409 | 189G 263G 271T 302_1C 302_2C 315_1C | 0 (1) | 263G 302_1C 315_1C | 0 (1) | Different motif |
| OXH-A-2 | 16517-160 | 16519C 73G 150T | 2 (0.0812) | 73G 150T | 2 (0.1958) | JP(16519C<>16519T) |
| SAE-A-1 | 16055-16218 | 16126C | 3.167 (0.045) | 16192T | 3 (0.1002) | Different motif |
| SFA-B-1 | 183-409 | 263G 302_1C 302_2C 315_1C | 15.333 (0) | 263G 302_1C 315_1C | 11.833 (0) | I(302_2C) |
| SFA-B-1 | 16517-334 | 55C 55_1A 263G 302_1C 302_2C 315_1C | 4.5 (0.0346) | 55C 55_1A 263G 302_1C 315_1C | 3.5 (0.109) | I(302_2C) |
| SFA-B-1 | 16055-16410 | 16253G | 0 (1) |  |  | Fragment excluded |
| SFS-A-1 | 16055-16218 | 16126C 16153A 16183T 16183_1C 16189C | 6 (0) | 16126C 16153A 16183_1C 16189C | 3 (0.0304) | CDS(16183T) |
| SFS-A-1 | 183-409 | 263G 302_2C 304T 306T 315_1C 332T 371T 381T | 32 (0) | 263G 302_2C 315_1C | 17.167 (0) | CDS(304T 306T 332T 371T 381T) |
| SHS-A-1 | 16517-160 | 16519C 73G 81A 152C | 2 (0.1554) | 16519C 73G 152C | 2 (0.2756) | CDS(81A) |
| SHS-A-1 | 16209-16410 | 16311C | 0 (1) | 16224C 16311C 16319A | 0 (1) | Different motif |
| SHS-A-1 | 16055-16410 | 16063C 16224C 16311C 16319A | 1 (1) | 16224C 16311C 16319A | 0 (1) | PMS(16063C) |
| SHS-A-1 | 16055-16218 | 16093C | 1.5 (0.2302) | CRS | 1 (0.8728) | Different motif |
| SHS-A-1 | 16517-334 | 16519C 73G 152C 263G 302_1C 315_1C | 4 (0.061) |  |  | Fragment excluded |
| SSG-A-1 | 16055-16410 | 16172C 16256T 16328T 16399G | 20 (0) | 16172C 16256T 16399G | 15.5 (0) | CDS(16328T) |
| SSG-A-3 | 16055-16410 | 16108T 16126C 16256T 16294T | 0 (1) | 16126C 16294T | 0 (1) | CDS(16108T 16256T) |
| SSG-A-4 | 16209-16410 | 16298C | 3.5 (0.0406) | 16261T | 3 (0.0294) | Different motif |
| SSJ-A-2 | 16055-16410 | 16126C 16294T 16296T 16304C | 0 (1) | 16172C 16256T 16399G | 0 (1) | CDS(16274A 16361A) |
| SSJ-A-2 | 183-409 | 263G 302_1C 315_1C | 3.333 (0.0154) | 263G 315_1C | 2 (0.221) | I(302_1C) |
| STB-A-1 | 16209-16410 | 16260T 16263C | 9 (0.0002) | 16263C | 7.5 (0) | CDS(16260T) |
| STH-A-1 | 16517-160 | 16519C 73G | 1 (0.5082) | 16519C 73G 152C | 1 (0.5056) | CDS(9A 35A 53A) |
| STH-A-1 | 16517-334 | 16519C 16565T 6T 73G 152C 189G 199C 204C 207A 250C 263G 295T 315_1C | 0 (1) | 16519C 73G 152C 189G 199C 204C 207A 250C 263G 315_1C | 0 (1) | CDS(16565T 6T 295T) |
| STK-A-1 | 16209-16410 | 16210G 16239T | 1 (1) | 16239T | 0 (1) | PMS(16210G) |
| STK-A-1 | 16055-16410 | 16080G 16129A 16239T | 1 (1) | 16129A 16239T | 0 (1) | PMS(16080G) |
| STK-A-2 | 16055-16410 | 16072T 16129A 16223T 16254G 16278T 16287T 16290T 16391A | 1 (0.4422) | 16129A 16223T 16391A | 0 (1) | CDS(16072T 16278T 16287T 16290T) |
| STT-A-2 | 16517-160 | 16519C 73G 128T 152C | 5 (0.0298) | 16519C 73G 152C | 3.5 (0.0298) | CDS(128T) |
| SUB-A-1 | 16055-16410 | 16093C 16183_3C 16189C 16270T | 12 (0) | 16093C 16189C 16270T | 7.333 (0) | I(16183_3C) |
| SUB-B-1 | 16055-16218 | 16093C 16189C 16190T 16193_1C | 9 (0.0004) | 16093C 16189C 16193_1C | 8 (0) | CDS(16190T) |
| SVE-A-1 | 16055-16410 | 16069T 16126C 16239T | 0 (1) | 16069T 16126C 16278T | 0 (1) | JP(16239T<>16278T) |
| SVK-A-1 | 16055-16410 | 16222A 16294T 16304C 16311C | 2.5 (0.1212) | 16129A 16223T 16391A | 0 (1) | CDS(16184T 16185T 16211T 16327T 16337T 16355T 16358T 16393T) |
| SVK-A-1 | 183-409 | 199C 204C 207A 250C 263G 298-347Del- 315_1C | 6.583 (0.0022) | 199C 204C 207A 250C 263G 315_1C | 4.833 (0.0322) | I(298-347Del-) |
| SYK-A-1 | 16055-16410 | 16067T 16083T 16085T 16183- 16184T 16185T 16189C 16223T 16267T 16278T | 0 (1) | 16183C 16189C 16223T 16278T | 0 (1) | CDS(16112T 16268T 16270T 16279T 16282T 16355T 16379T 16393T), I(16183_1C ) |
| SYR-A-1 | 16055-16218 | 16189C 16191T | 10 (0.001) | 16189C | 8 (0) | CDS(16191T) |
| SYR-B-1 | 16055-16410 | 16067T 16069T 16185T 16224C 16354T | 0 (1) |  |  | Fragment excluded |
| ÞSK-A-26 | 183-409 | 263G 315_1C | 6.167 (0.0012) | 242T 263G 295T 315_1C | 4.5 (0.0282) | Different motif |
| ÞSK-A-26 | 16517-160 | 16519C 73G | 9.5 (0) | 73G | 6 (0.0078) | Different motif |
| ÞSK-A-26 | 16209-16410 | CRS | 5.783 (0.0016) | 16222T 16261T | 3.833 (0.139) | Different motif |
| TMY-A-2 | 16209-16410 | 16239T 16290T 16291T 16304C | 19 (0) | 16304C | 13.5 (0) | CDS(16239T 16290T 16291T ) |
| VDP-A-3 | 183-409 | 268T 263G 315_1C | 14 (0.0002) | 263G 315_1C | 9 (0) | CDS(268T) |
| VDP-A-5 | 16517-409 | 16519C 263G 315_1C 328G | 1.5 (0.5058) | 16519C 263G 315_1C | 1 (1) | PMS(328G) |
| VDP-A-7 | 16055-16410 | 16298C 16311C 16325C 16327T | 0 (1) | CRS | 0 (1) | CDS(16095T) |
| VDS-A-1 | 16517-160 | 73G | 0 (1) | 16519C 73G | 0 (1) | CDS(16546T 6T 29T 31T 105T 110T 112T 141T) |
