# Supplementary material for: Sequences From First Settlers Reveal Rapid Evolution in Icelandic mtDNA Pool
Source: PLoS Genet. 2009 Jan 16;5(1):e1000343. doi: 10.1371/journal.pgen.1000343 (PMC2613751; doi:10.1371/journal.pgen.1000343)
Supplement: Table S4 — Results from replication amplifications performed in Reykjavik. (0.08 MB DOC) [file pgen.1000343.s004.doc]

Table S4. Results from replication amplifications performed in Reykjavik

| **Skeletal remains** | **No. of DNA extracts used for amplifications** | **Sequence fragment** | **Amplification (number of clone sequences)** | **Sequence assigned to fragment** | **Assigned sequence present in all amplifications** |
| --- | --- | --- | --- | --- | --- |
| BRE-A-1 | 1 | 16055-16218 | 1(12), 2(12) | 16069T 16126C 16147T | Yes |
| ASS-A-1 | 1 | 16055-16410 | 1(11), 2(1) | CRS | No |
| ASS-A-1 | 1 | 16517-160 | 1(5), 2(7) | 16519C | Yes |
| DAP-A-1 | 1 | 16517-160 | 1(3), 2(9) | 73G | Yes |
| DAP-A-1 | 1 | 16055-16218 | 1(12), 2(12) | CRS | Yes |
| BSE-A-1 | 1 | 16055-16410 | 1(11), 2(2) | 16069T 16126C | No |
| DAV-A-8 | 1 | 16209-16410 | 1(12), 2(31) | CRS | Yes |
| DAV-A-8 | 1 | 16517-160 | 1(6), 2(29) | 16519C | Yes |
| EFS-A-1 | 1 | 16209-16410 | 1(10), 2(31) | 16239T | Yes |
| FOV-A-1 | 1 | 183-334/409 | 1(1), 2(10) | 263G 302_1C 315_1C | Yes |
| GRS-A-2 | 1 | 16055-16410 | 1(12), 2(10) | 16256T | Yes |
| GRM-A-1 | 1 | 16517-160 | 1(10), 2(43) | 16519C 73G 152C | Yes |
| NUA-A-1 | 1 | 16209-16410 | 1(12), 2(11) | CRS | Yes |
| NUA-A-1 | 1 | 16517-160 | 1(9), 2(11) | 16519C 146C | Yes |
| MKL-A-1 | 1 | 16209-16410 | 1(12), 2(30) | 16223T 16286T | Yes |
| NÞR-A-1 | 1 | 16209-16410 | 1(7), 2(19) | 16224C 16311C 16320T | Yes |
| NÞR-A-1 | 1 | 16517-160 | 1(4), 2(22) | 16519C 73G 146C 152C | Yes |
| NÞR-A-2 | 1 | 16209-16410 | 1(12), 2(12) | 16224C 16249C 16311C | Yes |
| SSG-A-4 | 1 | 16055-16410 | 1(6), 2(5) | 16069T 16126C 16145A 16172C 16192T 16261T | No |
| SHS-A-1 | 1 | 183-334/409 | 1(10), 2(4), 3(7) | 263G 298- 315_1C | Yes |
| SYK-A-1 | 1 | 16055-16218 | 1(1), 2(12) | 16183C 16189C 16193_1C | Yes |
| ABH-A-1 | 1 | 16055-16218 | 1(12), 2(12) | CRS | Yes |
| GRM-A-1 | 1 | 16517-334/409 | 1(13), 2(8) | 16519C 73G 152C 195C 263G 315_1C | Yes |
| HVL-A-2 | 1 | 16517-160 | 1(8), 2(10) | 16519C 46C 152C | Yes |
| GRM-A-1 | 1 | 183-334/409 | 1(40), 2(36) | 195C 263G 315_1C | Yes |
| HSJ-A-1 | 2 | 16055-16218 | 1(7), 2(12) | CRS | Yes |
| HSJ-A-1 | 2 | 16209-16410 | 1(7), 2(4) | CRS | Yes |
| HSJ-A-1 | 2 | 16517-160 | 1(30), 2(12) | 16519C 46C 152C | Yes |
| HSJ-A-1 | 2 | 16517-334/409 | 1(32), 2(6) | 16519C 46C 152C 302_1C 315_1C | Yes |
| SSG-A-1 | 2 | 16055-16218 | 1(12), 2(12) | 16172C | Yes |
| SSG-A-1 | 2 | 16209-16410 | 1(11), 2(10) | 16256T 16399G | Yes |
| SSG-A-1 | 2 | 16517-160 | 1(8), 2(12) | 73G | Yes |
| SSG-A-1 | 2 | 16517-334/409 | 1(7), 2(3) | 73G 263G 302_1C 302_2C 315_1C | Yes |
| SSG-A-1 | 2 | 183-334/409 | 1(11), 2(11) | 263G 302_1C 302_2C 315_1C | Yes |
