# Supplementary material for: Sequences From First Settlers Reveal Rapid Evolution in Icelandic mtDNA Pool
Source: PLoS Genet. 2009 Jan 16;5(1):e1000343. doi: 10.1371/journal.pgen.1000343 (PMC2613751; doi:10.1371/journal.pgen.1000343)
Supplement: Table S5 — Results from replication amplifications performed in Barcelona. (0.05 MB DOC) [file pgen.1000343.s005.doc]

Table S5. Results from replication amplifications performed in Barcelona

| **Skeletal remains** | **Fragment** | **Sequence assigned to fragment based on clones from Reykjavik lab** | **Reykjavik sequence found among Barcelona clones** | **Number of clones** | **Barcelona clone sequence with greatest c-value** | **c_MAX_ (P-value)** |
| --- | --- | --- | --- | --- | --- | --- |
| FOV-A-1 (tooth 1) | 16055-16218 | 16111T | Yes | 8 | 16111T | 2 (0.55) |
| FOV-A-1 (tooth 2) | 16022-16218 | 16111T | Yes | 10 | 16111T | 10.5 (0) |
| FOV-A-1 (tooth 2) | 16185-16378 | 16311C | Yes | 11 | 16311C | 28.8 (0) |
| BRE-A-1 | 16072-16218 | 16126C | Yes | 4 | 16126C | 13.5 (0) |
| GRV-A-1 | 16209-16401 | 16223T 16249C 16391A | Yes | 6 | 16249C | 3.8 (0.112) |
| HBS-A-6 | 183-409 | 199C 204C 207A 250C 263G 315_1C | Yes | 7 | 199C 204C 207A 250C 263G 315_1C | 2 (0.461) |
| KHF-A-1 | 16209-16401 | 16224C 16234T 16270T 16311C | Yes | 7 | 16224C 16234T 16270T 16311C | 12 (0) |
| NNM-A-1 | 16209-16401 | 16235G 16291T | Yes | 5 | 16235G | 2.5 (0.128) |
| SHS-A-1 | 16209-16401 | 16224C 16311C 16319A | Yes | 9 | 16224C 16311C 16319A | 14 (0) |
| SUB-A-1 | 16209-16401 | 16270T | Yes | 4 | 16270T | 1 (1) |
| STK-A-1 | 16209-16401 | 16239T | No | 13 | 16224C 16311C 16320T | 9 (0) |
| STK-A-1 | 16517-160 | 16519C 152C | No | 8 | 16519C 73G 146C 152C | 4 (0.07) |
| UAM-B-1 | 16517-160 | 16519C 73G | Yes | 8 | 16519C 73G | 1 (0.632) |
| UAM-B-1 | 16209-16401 | 16291T 16294T 16296T | Yes | 8 | 16291T 16294T 16296T | 3 (0.189) |
| UAM-B-1 | 16055-16401 | 16126C 16291T 16294T 16296T | Yes | 2 | 16126C 16291T 16294T 16296T | 0 (1) |
| VDP-A-6 | 16517-160 | 16519C 146C | Yes | 6 | 16519C 146C | 1 (1) |
| VDP-A-6 | 16055-16218 | 16176T | Yes | 4 | 16176T | 1 (1) |
| VDP-A-6 | 16209-16401 | 16219G | Yes | 7 | 16219G 16305G | 1 (1) |
