# Supplementary material for: Sequences From First Settlers Reveal Rapid Evolution in Icelandic mtDNA Pool
Source: PLoS Genet. 2009 Jan 16;5(1):e1000343. doi: 10.1371/journal.pgen.1000343 (PMC2613751; doi:10.1371/journal.pgen.1000343)
Supplement: Table S6 — List of sampled skeletal remains with background information. (0.19 MB DOC) [file pgen.1000343.s006.doc]

Table S6. List of sampled skeletal remains with background information

| **Skeletal remains** | **Sex** | **Period** | **Year excavated** | **Removed from jaw during sampling** | **ToothType** | **Sequence motif for region 16055-16410** | **Sequence motif for region 16517-334** |
| --- | --- | --- | --- | --- | --- | --- | --- |
| ABH-A1 | female | Pre-Christian | 1893 | Yes | Molar |  |  |
| AEY-A1 | female | Pre-Christian | 1934 | Yes | Incisor |  |  |
| ASS-A1 | ? | Pre-Christian | 1945 | Yes | Premolar | CRS | 16519C 263G 315_1C |
| BAJ-A1 | male? | Pre-Christian | 1988 | No | Canine | 16192T 16223T 16325C | 16519C 73G 189G 195C 204C 207A 263G 315_1C |
| BBE-A1 | male? | Pre-Christian | 1937 | Yes | Premolar | 16129A | 16519C 263G 302_1C 315_1C |
| BRE-A1 | ? | Pre-Christian | 1937 | Yes | Molar | 16069T 16126C 16147T | 73G 185A 228A 263G 295T 302_1C 315_1C |
| BRV-A3 | ? | Pre-Christian | 1937 | Yes | Molar |  |  |
| BSE-A1 | ? | Pre-Christian | 1950 | Yes | Molar | 16069T 16126C | 73G 185A 228A 263G 295T 315_1C |
| DAP-A1 | ? | Pre-Christian | 1956 | Yes | Molar | 16355T 16356C | 73G 195C 263G 315_1C |
| DAV-A1 | male | Pre-Christian | 1909 | Yes | Molar | 16162G 16209C | 16519C 73G 263G 315_1C |
| DAV-A2 | female? | Pre-Christian | 1909 | Yes | Molar |  |  |
| DAV-A4 | male? | Pre-Christian | 1909 | No | Molar |  |  |
| DAV-A6 | female | Pre-Christian | 1909 | Yes | Molar |  |  |
| DAV-A7 | male? | Pre-Christian | 1909 | Yes | Canine | 16301T 16343G 16356C 16390A | 16519C 73G 150T 263G 315_1C |
| DAV-A8 | female | Pre-Christian | 1909 | No | Molar | CRS | 16519C 263G 315_1C |
| DAV-A9 | female? | Pre-Christian | 1909 | Yes | Canine | CRS | 16519C 263G 315_1C |
| DKS-A1 | male? | Pre-Christian | 1962 | Yes | Molar | 16192T 16239T 16256T 16270T 16399G | 73G 150T 263G 302_1C 315_1C |
| EFS-A1 | female? | Pre-Christian | 1916 | Yes | Molar |  |  |
| EIM-A1 | female | Pre-Christian | 1979 | No | Molar | CRS | 16519C 263G 315_1C |
| ENV-A1 | male? | Pre-Christian | 1935 | Yes | Molar | 16162G | 16519C 73G 263G 315_1C |
| EVS-A1 | male | Pre-Christian | 1956 | Yes | Molar | 16362C | 239C 263G 302_1C 315_1C |
| FOV-A1 | male | Pre-Christian | 1929 | Yes | Molar and Premolar | 16111T 16311C | 263G 302_1C 315_1C |
| FSS-A1 | female | Pre-Christian | 1960 | Yes | Premolar | 16356C | 16519C 73G 195C 263G 310C |
| GGH-A1 | male | Pre-Christian | 1934 | Yes | Molar | 16293G | 16519C 263G 315_1C |
| GRF-A1 |  | Pre-Christian | 1962 | Yes | Molar | 16069T 16126C | 73G 185A 228A 263G 295T 315_1C |
| GRM-A1 | male | Pre-Christian | 1937 | Yes | Molar | 16126C 16294T 16304C | 16519C 73G 152C 195C 263G 315_1C |
| GRS-A1 | male | Pre-Christian | 1967 | Yes | Premolar | 16093C 16224C 16311C | 16519C 73G 114T 263G 302_1C 315_1C |
| GRS-A2 | male | Pre-Christian | 1967 | Yes | Premolar | 16256T | 16519C 263G 302_1C 315_1C |
| GRV-A1 | male? | Pre-Christian | 1935 | Yes | Canine | 16129A 16223T 16249C 16391A | 16519C 73G 189G 199C 204C 250C 263G 315_1C |
| GTE-A1 | male | Pre-Christian | 1957 | Yes | Premolar | CRS | 263G 315_1C |
| GTE-A2 | male | Pre-Christian | 1957 | Yes | Molar |  |  |
| HBS-A6 | male | Pre-Christian | 1947 | Yes | Molar | 16129A 16223T 16391A | 16519C 73G 152C 199C 204C 207A 250C 263G 315_1C |
| HRK-A1 | male? | Pre-Christian | 1952 | No | Molar |  |  |
| HRK-A2 | female | Pre-Christian | 1952 | Yes | Molar | 16069T 16126C 16145A 16172C 16192T 16222T 16261T | 73G 242T 263G 295T 315_1C |
| HSJ-A1 | male | Pre-Christian | 1996 | Yes | Molar and Molar | CRS | 16519C 46C 152C 302_1C 315_1C |
| HVL-A2 | ? | Pre-Christian | 1932 | No | Molar |  |  |
| KHF-A1 | female | Pre-Christian | 1958 | Yes | Molar | 16224C 16234T 16270T 16311C | 16519C 73G 113T 263G 315_1C |
| KNS-A1 | male | Pre-Christian | 1932 | No | Molar | 16304C 16362C | 263G 302_1C 315_1C |
| KRE-A1 | male? | Pre-Christian | 1900 | Yes | Premolar | 16069T 16126C | 73G 228A 263G 295T 302_1C 315_1C |
| KVE-A1 | male | Pre-Christian | 1925 | Yes | Incisor |  |  |
| LKH-A1 | ? | Pre-Christian | 1969 | No | Molar | 16126C 16294T 16296T 16304C | 16519C 73G 204C 207A 263G 302_1C 315_1C |
| MKL-A1 | female? | Pre-Christian | 1936 | No | Canine | 16223T 16286T | 16519C 73G 143A 189G 192C 194T 195C 196C 204C 207A 263G 315_1C |
| MKR-A1 | male | Pre-Christian | 1989 | Yes | Premolar | 16224C 16311C | 16519C 73G 146C 152C 263G 315_1C |
| NNM-A1 | female? | Pre-Christian | 1981 | Yes | Molar | 16235G 16291T | 263G 302_1C 315_1C |
| NÞR-A1 | male | Pre-Christian | 1952 | No | Canine | 16224C 16311C 16320T | 16519C 73G 146C 152C 263G 315_1C |
| NÞR-A2 | female? | Pre-Christian | 1952 | Yes | Molar | 16224C 16249C 16311C | 16519C 73G 263G 302_1C 315_1C |
| NUA-A1 | female | Pre-Christian | 1915 | Yes | Molar | 16189C | 16519C 146C 263G 315_1C |
| ORE-A1 | male | Pre-Christian | 1966 | No | Canine | 16224C 16311C 16362C | 16519C 73G 263G 315_1C |
| OXH-A2 | male | Pre-Christian | 1962 | Yes | Molar | 16192T 16270T | 73G 150T 263G 315_1C |
| SAE-A1 | male? | Pre-Christian | 1917 | No | Molar |  |  |
| SBT-A1 | male | Pre-Christian | 1954 | No | Molar | CRS | 16519C 152C 263G 315_1C |
| SFA-A1 | female? | Pre-Christian | 1958 | No | Molar |  |  |
| SFA-B1 | female? | Pre-Christian | 1962 | Yes | Molar | CRS | 55C 55_1A 263G 302_1C 315_1C |
| SFS-A1 | female? | Pre-Christian | 1901 | Yes | Premolar | 16126C 16153A 16183_1C 16189C 16294T 16390A | 16519C 41T 73G 150T 263G 302_1C 315_1C |
| SHS-A1 | male? | Pre-Christian | 1956 | Yes | Premolar | 16224C 16311C 16319A | 16519C 73G 152C 263G 298- 315_1C |
| SSG-A1 | male | Pre-Christian | 1947 | Yes | Canine and Premolar | 16172C 16256T 16399G | 73G 263G 302_1C 302_2C 315_1C |
| SSG-A2 | male | Pre-Christian | 1947 | No | Premolar | 16069T 16126C | 73G 185A 189G 263G 295T 315_1C |
| SSG-A3 | male | Pre-Christian | 1947 | No | Molar | 16126C 16294T | 16519C 73G 152C 263G 302_1C 315_1C |
| SSG-A4 | female? | Pre-Christian | 1947 | No | Premolar | 16069T 16126C 16145A 16172C 16192T 16261T | 73G 242T 263G 295T 315_1C |
| SSJ-A2 | male | Pre-Christian | 1949 | Yes | Incisor | 16172C 16256T 16399G | 73G 263G 302_1C 315_1C |
| SSS-A1 |  | Unknown | 1993 | Yes | Molar | CRS | 16519C 263G 315_1C |
| SSV-A1 |  | Pre-Christian | 1983 | Yes | Molar | 16304C 16305G | 263G 315_1C |
| STB-A1 | male | Pre-Christian | 1933 | Yes | Incisor | 16263C | 16519C 263G 315_1C |
| STH-A1 | male | Pre-Christian | 1935 | Yes | Premolar | 16129A 16223T 16362C 16391A | 16519C 73G 152C 189G 199C 204C 207A 250C 263G 315_1C |
| STH-A2 | male | Pre-Christian | 1935 | Yes | Incisor |  |  |
| STK-A1 | male? | Pre-Christian | 1933 | No | Molar |  |  |
| STK-A2 | female? | Pre-Christian | 1933 | Yes | Premolar | 16129A 16223T 16391A | 16519C 73G 146C 199C 204C 250C 263G 315_1C |
| STT-A1 | male | Pre-Christian | 1952 | Yes | Premolar |  |  |
| STT-A2 | male | Pre-Christian | 1952 | Yes | Molar | 16356C | 16519C 73G 152C 195C 263G 315_1C |
| STT-A4 | ? | Pre-Christian | 1952 | Yes | Incisor |  |  |
| SUB-A1 | male | Pre-Christian | 1947 | Yes | Premolar | 16093C 16189C 16270T | 73G 150T 263G 315_1C |
| SUB-B1 | male? | Pre-Christian | 1975 | Yes | Premolar | 16093C 16183_1C 16189C 16270T | 73G 150T 263G 315_1C |
| SVE-A1 | male | Pre-Christian | 1968 | Yes | Premolar | 16069T 16126C 16278T | 16519C 73G 150T 152C 263G 295T 315_1C |
| SVK-A1 | male | Pre-Christian | 1961 | Yes | Incisor | 16129A 16223T 16391A | 16519C 73G 152C 199C 204C 207A 250C 263G 315_1C |
| SYK-A1 | male | Pre-Christian | 1963 | Yes | Incisor | 16183C 16189C 16223T 16278T | 16519C 73G 153G 195C 225A 226C 263G 315_1C |
| SYR-A1 | female | Pre-Christian | 1936 | No | Molar | 16189C 16298C | 72C 263G 302_1C 315_1C |
| SYR-B1 | female? | Pre-Christian | 1940 | Yes | Molar | 16224C 16311C | 16519C 73G 146C 152C 263G 315_1C |
| TGS-A1 | male | Unknown | 1981 | Yes | Molar | 16126C 16153A 16294T | 16519C 41T 73G 150T 263G 302_1C 302_2C 315_1C |
| ÞLS-A1 | male? | Pre-Christian | 1948 | Yes | Premolar | 16298C | 72C 263G 302_1C 302_2C 315_1C |
| THS-A1 | ? | Pre-Christian | 1880 | No | Molar |  |  |
| ÞSK-A1 | female | Pre-1104 | 1939 | Yes | Molar | 16166G 16224C 16290T 16311C | 16519C 73G 263G 280G 315_1C |
| ÞSK-A26 | male | Pre-1104 | 1939 | Yes | Molar | 16069T 16126C 16145A 16172C 16192T 16222T 16261T | 73G 242T 263G 295T 315_1C |
| TMY-A1 | male? | Pre-Christian | 1935 | Yes | Premolar |  |  |
| TMY-A2 | male? | Pre-Christian | 1935 | Yes | Molar |  |  |
| UAM-B1 | male? | Pre-Christian | 1961 | Yes | Molar | 16126C 16291T 16294T 16296T | 16519C 73G 263G 315_1C |
| VDP-A3 | male | Pre-Christian | 1964 | Yes | Premolar | 16224C 16311C | 16519C 73G 146C 152C 263G 315_1C |
| VDP-A5 | female | Pre-Christian | 1964 | Yes | Premolar | CRS | 16519C 263G 315_1C |
| VDP-A6 | male | Pre-Christian | 1964 | Yes | Molar | 16176T 16219G | 16519C 146C 257G 263G 315_1C |
| VDP-A7 | male | Pre-Christian | 1964 | Yes | Premolar | CRS | 263G 315_1C |
| VDS-A1 | ? | Pre-Christian | 1927 | No | Molar | 16311C | 16519C 73G 263G 302_1C 315_1C |
| VSL-A1 | ? | Pre-Christian | 1946 | Yes | Premolar |  |  |
| YGS-A2 | female | Pre-Christian | 1956 | No | Molar | 16126C 16294T 16296T 16304C | 16519C 73G 263G 315_1C |
| YGS-B1 | male | Pre-Christian | 1958 | Yes |  | 16069T 16126C | 73G 228A 263G 295T 315_1C |
| YGS-B2 | male? | Pre-Christian | 1958 | Yes | Molar | 16069T 16126C | 73G 228A 263G 295T 315_1C |
| YGS-B5 | female? | Pre-Christian | 1958 | No | Molar |  |  |
