# Supplementary material for: Sequences From First Settlers Reveal Rapid Evolution in Icelandic mtDNA Pool
Source: PLoS Genet. 2009 Jan 16;5(1):e1000343. doi: 10.1371/journal.pgen.1000343 (PMC2613751; doi:10.1371/journal.pgen.1000343)
Supplement: Table S7 — Oligonucleotide primer sequences. (0.04 MB DOC) [file pgen.1000343.s007.doc]

Table S7. Oligonucleotide primer sequences

| **Primer** | **Sequence** | **Reykjavik** | **Barcelona** |
| --- | --- | --- | --- |
| L16055 | GAAGCAGATTTGGGTACCAC | X |  |
| L16209 | CCCCATGCTTACAAGCAAGT | X | X |
| L16517 | CATCTGGTTCCTACTTCAGG | X | X |
| L183 | ATCGCACCTACGTTCAATATTACA | X | X |
| H16410 | GCGGGATATTGATTTCACGG | X |  |
| H16218 | TGTGTGATAGTTGAGGGTTG | X | X |
| H409 | CTGTTAAAAGTGCATACCGCC | X | X |
| H160 | TGTAATATTGAACGTAGGTGCGAT | X | X |
| H334 | TGGGGTTTGGCAGAGATGTGTTTA | X |  |
| L16022 | AGATTCTAATTTAAACTATTCCTCT |  | X |
| H16401 | TGATTTCACGGAGGATGGTG |  | X |
| L16185 | AACCCAATCCACATCAAAACC |  | X |
| H16378 | CAAGGGACCCCTATCTGAGG |  | X |
