# Supplementary material for: Sequences From First Settlers Reveal Rapid Evolution in Icelandic mtDNA Pool
Source: PLoS Genet. 2009 Jan 16;5(1):e1000343. doi: 10.1371/journal.pgen.1000343 (PMC2613751; doi:10.1371/journal.pgen.1000343)
Supplement: Table S8 — Primer pairs used for DNA amplifications. (0.04 MB DOC) [file pgen.1000343.s008.doc]

Table S8. Primer pairs used for DNA amplifications

| ***Forward Primer*** | ***Reverse Primer*** | ***Amplicon length*** | ***Annealing Temp (°C)*** | ***Reykjavik*** | ***Barcelona*** |
| --- | --- | --- | --- | --- | --- |
| L16055 | H16410 | 394 | 54.5 | X |  |
| L16055 | H16218 | 202 | 53 | X | X |
| L16209 | H16410 | 240 | 55.5 | X |  |
| L16517 | H409 | 501 | 55 | X |  |
| L16517 | H160 | 255 | 52 | X | X |
| L183 | H409 | 270 | 53.5 | X | X |
| L16517 | H334 | 429 | 53 | X |  |
| L183 | H334 | 198 | 52.5 | X |  |
| L16185 | H16378 | 233 | 52 |  | X |
| L16055 | H16401 | 385 | 50 |  | X |
| L16022 | H16218 | 240 | 50 |  | X |
| L16209 | H16401 | 231 | 52 |  | X |
