# Supplementary material for: Sequences From First Settlers Reveal Rapid Evolution in Icelandic mtDNA Pool
Source: PLoS Genet. 2009 Jan 16;5(1):e1000343. doi: 10.1371/journal.pgen.1000343 (PMC2613751; doi:10.1371/journal.pgen.1000343)
Supplement: Table S9 — Rules for haplogroup classification of sequences. (0.30 MB DOC) [file pgen.1000343.s009.doc]

Table S9. Rules for haplogroup classification of sequences

|  |  | **Values added to haplogroup scores when rule is true** | | | | | | | | | | | | | |
| --- | --- | --- | --- | --- | --- | --- | --- | --- | --- | --- | --- | --- | --- | --- | --- |
| **Fragment** | **Rule (! = not)** | **H** | **I** | **J** | **K** | **T** | **U5** | **U3** | **U2** | **U4** | **V** | **W** | **X** | **Z** | **C** |
| 16055-16218 | CRS | 1 | 0 | 0 | 1 | 0 | 1 | 1 | 0 | 1 | 1 | 1 | 0 | 0 | 1 |
| 16055-16218 | 16129A | 1 | 1 | 0 | 0 | 0 | 0 | 0 | -1 | 0 | 0 | 0 | 0 | 1 | 0 |
| 16055-16218 | 16129!A | 0 | -5 | 0 | 0 | 0 | 0 | 0 | 0 | 0 | 0 | 0 | 0 | 0 | 0 |
| 16055-16218 | 16069T 16126C | -1 | -1 | 5 | -1 | -1 | -1 | -1 | -1 | -1 | -1 | -1 | -1 | -1 | -1 |
| 16055-16218 | 16069!T 16126C | 1 | 0 | 0 | 1 | 1 | 0 | 0 | 0 | 0 | 1 | 0 | 1 | 0 | 0 |
| 16055-16218 | 16069!T 16126!C | 0 | 0 | -5 | 0 | 0 | 0 | 0 | 0 | 0 | 0 | 0 | 0 | 0 | 0 |
| 16055-16218 | 16126!C | 0 | 0 | 0 | 0 | -5 | 0 | 0 | 0 | 0 | 0 | 0 | 0 | 0 | 0 |
| 16055-16218 | 16051G | 1 | 0 | 0 | 0 | 0 | 0 | 0 | 1 | 0 | 0 | 0 | 0 | 0 | 0 |
| 16055-16218 | 16192T | 1 | 0 | 1 | 0 | 1 | 1 | 0 | 0 | 0 | 0 | 1 | 0 | 0 | 0 |
| 16055-16218 | 16189C | 1 | 0 | 0 | 1 | 1 | 1 | 1 | 1 | 1 | 1 | 0 | 1 | 0 | 0 |
| 16055-16218 | 16189!C | 0 | 0 | 0 | 0 | 0 | 0 | 0 | 0 | 0 | 0 | 0 | -5 | 0 | 0 |
| 16055-16218 | 16172C | 1 | 1 | 1 | 0 | 1 | 1 | 0 | 0 | 0 | 0 | 0 | 0 | 0 | 0 |
| 16055-16218 | 16129C | -1 | -1 | -1 | -1 | -1 | -1 | -1 | 5 | -1 | -1 | -1 | -1 | -1 | -1 |
| 16055-16218 | 16129!C | 0 | 0 | 0 | 0 | 0 | 0 | 0 | -5 | 0 | 0 | 0 | 0 | 0 | 0 |
| 16209-16410 | CRS | 1 | -1 | 1 | -1 | -1 | -1 | -1 | 1 | -1 | 1 | -1 | -1 | -1 | -1 |
| 16209-16410 | 16356C | 1 | 0 | 0 | 0 | 0 | 0 | 1 | 0 | 1 | 0 | 0 | 0 | 0 | 0 |
| 16209-16410 | 16356!C | 0 | 0 | 0 | 0 | 0 | 0 | 0 | 0 | -5 | 0 | 0 | 0 | 0 | 0 |
| 16209-16410 | 16343G | -1 | -1 | -1 | -1 | -1 | -1 | 5 | -1 | -1 | -1 | -1 | -1 | -1 | -1 |
| 16209-16410 | 16343!G | 0 | 0 | 0 | 0 | 0 | 0 | -5 | 0 | 0 | 0 | 0 | 0 | 0 | 0 |
| 16209-16410 | 16304C | 1 | 1 | 0 | 0 | 1 | 1 | 0 | 0 | 0 | 0 | 0 | 0 | 0 | 0 |
| 16209-16410 | 16298C | 1 | 0 | 0 | 0 | 1 | 0 | 0 | 0 | 0 | 1 | 0 | 0 | 1 | 1 |
| 16209-16410 | 16294T | 1 | 0 | 0 | 0 | 1 | 1 | 0 | 0 | 0 | 0 | 0 | 0 | 0 | 0 |
| 16209-16410 | 16294!T | 0 | 0 | 0 | 0 | -5 | 0 | 0 | 0 | 0 | 0 | 0 | 0 | 0 | 0 |
| 16209-16410 | 16292T | 1 | 0 | 0 | 0 | 1 | 1 | 0 | 0 | 0 | 0 | 1 | 0 | 0 | 0 |
| 16209-16410 | 16270T | 1 | 0 | 0 | 1 | 0 | 1 | 0 | 0 | 0 | 0 | 0 | 0 | 0 | 0 |
| 16209-16410 | 16256T 16270T | -1 | -1 | -1 | -1 | -1 | 5 | -1 | -1 | -1 | -1 | -1 | -1 | -1 | -1 |
| 16209-16410 | 16256!T 16270!T 16325!C | 0 | 0 | 0 | 0 | 0 | -5 | 0 | 0 | 0 | 0 | 0 | 0 | 0 | 0 |
| 16209-16410 | 16256T | 1 | 0 | 0 | 0 | 0 | 1 | 0 | 0 | 0 | 0 | 0 | 0 | 0 | 0 |
| 16209-16410 | 16224C 16311C | -1 | -1 | -1 | 5 | -1 | -1 | -1 | -1 | -1 | -1 | -1 | -1 | -1 | -1 |
| 16209-16410 | 16224!C 16311!C | 0 | 0 | 0 | -5 | 0 | 0 | 0 | 0 | 0 | 0 | 0 | 0 | 0 | 0 |
| 16209-16410 | 16223T 16391A | -1 | 5 | -1 | -1 | -1 | -1 | -1 | -1 | -1 | -1 | -1 | -1 | -1 | -1 |
| 16209-16410 | 16223!T 16391!A | 0 | -5 | 0 | 0 | 0 | 0 | 0 | 0 | 0 | 0 | 0 | 0 | 0 | 0 |
| 16209-16410 | 16223T 16278T | -1 | -1 | -1 | -1 | -1 | -1 | -1 | -1 | -1 | -1 | -1 | 5 | -1 | -1 |
| 16209-16410 | 16223!T 16278!T | 0 | 0 | 0 | 0 | 0 | 0 | 0 | 0 | 0 | 0 | 0 | -5 | 0 | 0 |
| 16209-16410 | 16223T | 0 | 1 | 0 | 0 | 0 | 0 | 0 | 0 | 0 | 0 | 1 | 1 | 1 | 0 |
| 16209-16410 | 16223!T | 0 | -1 | 0 | 0 | 0 | 0 | 0 | 0 | 0 | 0 | -1 | -1 | -1 | 0 |
| 16209-16410 | 16223T 16292T | -1 | -1 | -1 | -1 | -1 | -1 | -1 | -1 | -1 | -1 | 5 | -1 | -1 | -1 |
| 16209-16410 | 16223!T 16292!T | 0 | 0 | 0 | 0 | 0 | 0 | 0 | 0 | 0 | 0 | -5 | 0 | 0 | 0 |
| 16209-16410 | 16223T 16298C | -1 | -1 | -1 | -1 | -1 | -1 | -1 | -1 | -1 | -1 | -1 | -1 | 5 | 5 |
| 16209-16410 | 16223!T 16298!C | 0 | 0 | 0 | 0 | 0 | 0 | 0 | 0 | 0 | 0 | 0 | 0 | -5 | -5 |
| 16055-16410 | CRS | 5 | -1 | -1 | -1 | -1 | -1 | -1 | -1 | -1 | 5 | -1 | -1 | -1 | -1 |
| 16055-16410 | 16126C 16294T | -1 | -1 | -1 | -1 | 5 | -1 | -1 | -1 | -1 | -1 | -1 | -1 | -1 | -1 |
| 16055-16410 | 16069T 16126C | -1 | -1 | 5 | -1 | -1 | -1 | -1 | -1 | -1 | -1 | -1 | -1 | -1 | -1 |
| 16055-16410 | 16129A 16223T 16391A | -1 | 5 | -1 | -1 | -1 | -1 | -1 | -1 | -1 | -1 | -1 | -1 | -1 | -1 |
| 16055-16410 | 16224C 16311C | -1 | -1 | -1 | 5 | -1 | -1 | -1 | -1 | -1 | -1 | -1 | -1 | -1 | -1 |
| 16055-16410 | 16298C | 1 | 0 | 0 | 0 | 1 | 0 | 0 | 0 | 0 | 1 | 0 | 0 | 1 | 1 |
| 16055-16410 | 16189C 16223T 16278T | -1 | -1 | -1 | -1 | -1 | -1 | -1 | -1 | -1 | -1 | -1 | 5 | -1 | -1 |
| 16055-16410 | 16256T 16270T | -1 | -1 | -1 | -1 | -1 | 5 | -1 | -1 | -1 | -1 | -1 | -1 | -1 | -1 |
| 16055-16410 | 16223T 16292T | -1 | -1 | -1 | -1 | -1 | -1 | -1 | -1 | -1 | -1 | 5 | -1 | -1 | -1 |
| 16055-16410 | 16126T 16304C | 1 | 0 | 0 | 0 | 0 | 0 | 0 | 0 | 0 | 0 | 0 | 0 | 0 | 0 |
| 16055-16410 | 16192T 16256T | -1 | -1 | -1 | -1 | -1 | 5 | -1 | -1 | -1 | -1 | -1 | -1 | -1 | -1 |
| 16055-16410 | 16192T 16270T | -1 | -1 | -1 | -1 | -1 | 5 | -1 | -1 | -1 | -1 | -1 | -1 | -1 | -1 |
| 16055-16410 | 16224!C 16311!C | 0 | 0 | 0 | -5 | 0 | 0 | 0 | 0 | 0 | 0 | 0 | 0 | 0 | 0 |
| 16055-16410 | 16069!T 16126!C | 0 | 0 | -5 | 0 | 0 | 0 | 0 | 0 | 0 | 0 | 0 | 0 | 0 | 0 |
| 16055-16410 | 16126!C 16294!T | 0 | 0 | 0 | 0 | -5 | 0 | 0 | 0 | 0 | 0 | 0 | 0 | 0 | 0 |
| 16055-16410 | 16129!A 16223!T 16391!A | 0 | -5 | 0 | 0 | 0 | 0 | 0 | 0 | 0 | 0 | 0 | 0 | 0 | 0 |
| 16055-16410 | 16189!C 16223!T 16278!T | 0 | 0 | 0 | 0 | 0 | 0 | 0 | 0 | 0 | 0 | 0 | -5 | 0 | 0 |
| 16055-16410 | 16189!C 16256!T 16270!T | 0 | 0 | 0 | 0 | 0 | -5 | 0 | 0 | 0 | 0 | 0 | 0 | 0 | 0 |
| 16055-16410 | 16223!T 16292!T | 0 | 0 | 0 | 0 | 0 | 0 | 0 | 0 | 0 | 0 | -5 | 0 | 0 | 0 |
| 16055-16410 | 16223!T 16298!C | 0 | 0 | 0 | 0 | 0 | 0 | 0 | 0 | 0 | 0 | 0 | 0 | -5 | -5 |
| 16055-16410 | 16223!T | 0 | -1 | 0 | 0 | 0 | 0 | 0 | 0 | 0 | 0 | -1 | -1 | -1 | -1 |
| 16055-16410 | 16356C | 1 | 0 | 0 | 0 | 0 | 0 | 1 | 0 | 1 | 0 | 0 | 0 | 0 | 0 |
| 16055-16410 | 16356!C | 0 | 0 | 0 | 0 | 0 | 0 | 0 | 0 | -5 | 0 | 0 | 0 | 0 | 0 |
| 16055-16410 | 16343G | -1 | -1 | -1 | -1 | -1 | -1 | 5 | -1 | -1 | -1 | -1 | -1 | -1 | -1 |
| 16055-16410 | 16343!G | 0 | 0 | 0 | 0 | 0 | 0 | -5 | 0 | 0 | 0 | 0 | 0 | 0 | 0 |
| 16055-16410 | 16129C | -1 | -1 | -1 | -1 | -1 | -1 | -1 | 5 | -1 | -1 | -1 | -1 | -1 | -1 |
| 16055-16410 | 16129!C | 0 | 0 | 0 | 0 | 0 | 0 | 0 | -5 | 0 | 0 | 0 | 0 | 0 | 0 |
| 16055-16410 | 16223T 16298C | -1 | -1 | -1 | -1 | -1 | -1 | -1 | -1 | -1 | -1 | -1 | -1 | 5 | 5 |
| 16517-160 | CRS | 5 | -1 | -1 | -1 | -1 | -1 | -1 | -1 | -1 | -1 | -1 | -1 | -1 | -1 |
| 16517-160 | 73G 153G | -1 | -1 | -1 | -1 | -1 | -1 | -1 | -1 | -1 | -1 | -1 | 5 | -1 | -1 |
| 16517-160 | 73G 153!G | 0 | 0 | 0 | 0 | 0 | 0 | 0 | 0 | 0 | 0 | 0 | -5 | 0 | 0 |
| 16517-160 | 73G | 1 | 1 | 1 | 1 | 1 | 1 | 1 | 1 | 1 | 0 | 1 | 1 | 1 | 1 |
| 16517-160 | 73A | 5 | -1 | -1 | -1 | -1 | -1 | -1 | -1 | -1 | 5 | -1 | -1 | -1 | -1 |
| 16517-160 | 72C 73A | -1 | -1 | -1 | -1 | -1 | -1 | -1 | -1 | -1 | 5 | -1 | -1 | -1 | -1 |
| 16517-160 | 72!C 73A | 0 | 0 | 0 | 0 | 0 | 0 | 0 | 0 | 0 | -5 | 0 | 0 | 0 | 0 |
| 16517-160 | 73G 146C 152C | 0 | 0 | 0 | 1 | 1 | 0 | 0 | 0 | 1 | 0 | 0 | 0 | 0 | 0 |
| 16517-160 | 73A 146C 152C | 1 | 0 | 0 | 0 | 0 | 0 | 0 | 0 | 0 | 0 | 0 | 0 | 0 | 0 |
| 16517-160 | 73G 150T | 1 | 0 | 1 | 1 | 1 | 1 | 1 | 0 | 0 | 0 | 0 | 0 | 0 | 0 |
| 16517-160 | 16519C 73A 152C | 5 | -1 | -1 | -1 | -1 | -1 | -1 | -1 | -1 | 0 | -1 | -1 | -1 | -1 |
| 16517-160 | 16519C 73G 152C | 1 | 1 | 1 | 1 | 1 | 0 | 0 | 1 | 1 | 0 | 0 | 0 | 1 | 0 |
| 16517-160 | 16519C 73G 150T | 0 | 0 | 1 | 1 | 1 | 1 | 1 | 0 | 0 | 0 | 0 | 0 | 0 | 0 |
| 16517-160 | 16519C 73G 146C | 0 | 0 | 0 | 1 | 0 | 0 | 0 | 0 | 0 | 0 | 0 | 0 | 0 | 0 |
| 16517-160 | 73G 150T 152C | 0 | 0 | 1 | 1 | 0 | 1 | 0 | 0 | 0 | 0 | 0 | 0 | 0 | 0 |
| 183-334 | CRS | 5 | -1 | -1 | -1 | -1 | -1 | -1 | -1 | -1 | -1 | -1 | -1 | -1 | -1 |
| 183-334 | 263A | 5 | 0 | 0 | 0 | 0 | 0 | 0 | 0 | 0 | 0 | 0 | 0 | 0 | 0 |
| 183-334 | 295T | -1 | -1 | 5 | -1 | -1 | -1 | -1 | -1 | -1 | -1 | -1 | -1 | -1 | -1 |
| 183-334 | 295!T | 0 | 0 | -5 | 0 | 0 | 0 | 0 | 0 | 0 | 0 | 0 | 0 | 0 | 0 |
| 183-334 | 199C 250C | -1 | 5 | -1 | -1 | -1 | -1 | -1 | -1 | -1 | -1 | -1 | -1 | -1 | -1 |
| 183-334 | 199!C 250!C | 0 | -5 | 0 | 0 | 0 | 0 | 0 | 0 | 0 | 0 | 0 | 0 | 0 | 0 |
| 183-334 | 189G | 1 | 1 | 1 | 1 | 0 | 0 | 0 | 0 | 0 | 1 | 1 | 0 | 0 | 0 |
| 183-334 | 189!G | 0 | 0 | 0 | 0 | 0 | 0 | 0 | 0 | 0 | 0 | -5 | 0 | 0 | 0 |
| 183-334 | 217C | 0 | 0 | 0 | 0 | 0 | 1 | 0 | 1 | 0 | 0 | 0 | 0 | 0 | 0 |
| 183-334 | 249- | -1 | -1 | -1 | -1 | -1 | -1 | -1 | -1 | -1 | -1 | -1 | -1 | 5 | 5 |
| 183-334 | 249!- | 0 | 0 | 0 | 0 | 0 | 0 | 0 | 0 | 0 | 0 | 0 | 0 | -5 | -5 |
| 183-334 | 204C | 1 | 1 | 0 | 0 | 1 | 1 | 0 | 0 | 0 | 0 | 1 | 0 | 0 | 0 |
| 183-334 | 225A | 0 | 0 | 0 | 0 | 0 | 0 | 0 | 0 | 0 | 0 | 0 | 1 | 0 | 0 |
| 16517-334 | CRS | 5 | -1 | -1 | -1 | -1 | -1 | -1 | -1 | -1 | -1 | -1 | -1 | -1 | -1 |
| 16517-334 | 73G 295T | -1 | -1 | 5 | -1 | -1 | -1 | -1 | -1 | -1 | -1 | -1 | -1 | -1 | -1 |
| 16517-334 | 73G 295!T | 0 | 0 | -5 | 0 | 0 | 0 | 0 | 0 | 0 | 0 | 0 | 0 | 0 | 0 |
| 16517-334 | 73G 249- | -1 | -1 | -1 | -1 | -1 | -1 | -1 | -1 | -1 | -1 | -1 | -1 | 5 | 5 |
| 16517-334 | 73G 249!- | 0 | 0 | 0 | 0 | 0 | 0 | 0 | 0 | 0 | 0 | 0 | 0 | -5 | -5 |
| 16517-334 | 73G 199C 250C | -1 | 5 | -1 | -1 | -1 | -1 | -1 | -1 | -1 | -1 | -1 | -1 | -1 | -1 |
| 16517-334 | 73G 199!C 250!C | 0 | -5 | 0 | 0 | 0 | 0 | 0 | 0 | 0 | 0 | 0 | 0 | 0 | 0 |
| 16517-334 | 73G 189G | 1 | 1 | 1 | 1 | 0 | 0 | 0 | 0 | 0 | 0 | 1 | 0 | 0 | 0 |
| 16517-334 | 73G 189G 204C | -1 | 5 | -1 | -1 | -1 | -1 | -1 | -1 | -1 | -1 | 5 | -1 | -1 | -1 |
| 16517-334 | 73G 189!G 204!C | 0 | 0 | 0 | 0 | 0 | 0 | 0 | 0 | 0 | 0 | -5 | 0 | 0 | 0 |
| 16517-334 | 73G 217C | 0 | 0 | 0 | 0 | 0 | 1 | 0 | 1 | 0 | 0 | 0 | 0 | 0 | 0 |
| 16517-334 | 73A 263!G | 5 | 0 | 0 | 0 | 0 | 0 | 0 | 0 | 0 | 0 | 0 | 0 | 0 | 0 |
| 16517-334 | 73G 153G 225A | -1 | -1 | -1 | -1 | -1 | -1 | -1 | -1 | -1 | -1 | -1 | 5 | -1 | -1 |
| 16517-334 | 73G 153!G 225!A | 0 | 0 | 0 | 0 | 0 | 0 | 0 | 0 | 0 | 0 | 0 | -5 | 0 | 0 |
| 16517-334 | 73G | 1 | 1 | 1 | 1 | 1 | 1 | 1 | 1 | 1 | 0 | 1 | 1 | 1 | 1 |
| 16517-334 | 73A | 5 | -1 | -1 | -1 | -1 | -1 | -1 | -1 | -1 | 5 | -1 | -1 | -1 | -1 |
| 16517-334 | 72C 73A | -1 | -1 | -1 | -1 | -1 | -1 | -1 | -1 | -1 | 5 | -1 | -1 | -1 | -1 |
| 16517-334 | 72!C 73A | 0 | 0 | 0 | 0 | 0 | 0 | 0 | 0 | 0 | -5 | 0 | 0 | 0 | 0 |
| 16517-334 | 73G 146C 152C | 0 | 0 | 0 | 1 | 1 | 0 | 0 | 0 | 1 | 0 | 0 | 0 | 0 | 0 |
| 16517-334 | 73A 146C 152C | 1 | 0 | 0 | 0 | 0 | 0 | 0 | 0 | 0 | 0 | 0 | 0 | 0 | 0 |
| 16517-334 | 73G 150T | 1 | 0 | 1 | 1 | 1 | 1 | 1 | 0 | 0 | 0 | 0 | 0 | 0 | 0 |
| 16517-334 | 16519C 73A 152C | 5 | -1 | -1 | -1 | -1 | -1 | -1 | -1 | -1 | 0 | -1 | -1 | -1 | -1 |
| 16517-334 | 16519C 73G 152C | 1 | 1 | 1 | 1 | 1 | 0 | 0 | 1 | 1 | 0 | 0 | 0 | 0 | 0 |
| 16517-334 | 16519C 73G 150T | 0 | 0 | 1 | 1 | 1 | 1 | 1 | 0 | 0 | 0 | 0 | 0 | 0 | 0 |
| 16517-334 | 16519C 73G 146C | 0 | 0 | 0 | 1 | 0 | 0 | 0 | 0 | 0 | 0 | 0 | 0 | 0 | 0 |
| 16517-334 | 73G 150T 152C | 0 | 0 | 1 | 1 | 0 | 1 | 0 | 0 | 0 | 0 | 0 | 0 | 0 | 0 |
| 16517-334 | 73G 204C | 1 | 1 | 0 | 0 | 0 | 1 | 0 | 0 | 0 | 0 | 1 | 0 | 0 | 0 |
