# Supplementary material for: Sequences From First Settlers Reveal Rapid Evolution in Icelandic mtDNA Pool
Source: PLoS Genet. 2009 Jan 16;5(1):e1000343. doi: 10.1371/journal.pgen.1000343 (PMC2613751; doi:10.1371/journal.pgen.1000343)
Supplement: Table S10 — Published mtDNA sequences used in comparative analyses (sites 16055-16391). (0.13 MB DOC) [file pgen.1000343.s010.doc]

Table S10. Published mtDNA sequences used in comparative analyses (sites 16055-16391)

| **Region** | **Populations and references** |
| --- | --- |
| Iceland | Icelanders[1,2] |
| North Atlantic Islands | Faroese[3], Islanders of Skye[4], Orkney Islanders[4], Shetlanders[5], Western Islanders[4] |
| Mainland Scotland and Ireland | Irish[6,7], Scottish[4] |
| Scandinavia | Norwegian[4,8], Swedish[9] |
| Northwest Europe | Austrian[10,11], German[12-16], Swiss[17,18] |
| Northeast Europe | Estonian[9], Finns[18,19], Latvian[9], Lithuanian[9] |
| Southwest Europe | Azores[20], French[21-23], Italian[24-27], Madeiran[20], Sardinian[28], Sicillian[29] |
| Eastern Europe | Czech[30], Poles[19,31], Russian[31], Slovenian[32] |
| European Russia | Karelians[9], Russian[33,34], Ukranian[34] |
| Southeast Europe | Bulgarians[35], Yugoslavian[36] |
| Northwest Africa | Berber[37], Canary Islanders[38], Mauritanian[37], Moroccan[37], Saharan[37] |
| Near East | Middle Eastern[28], Saudi-Arabian[39], Turks[35], Yemeni[40] |

**References for Table S10**

1. Helgason A, Nicholson G, Stefansson K, Donnelly P (2003) A reassessment of genetic diversity in Icelanders: strong evidence from multiple loci for relative homogeneity caused by genetic drift. Ann Hum Genet 67: 281-297.

2. Helgason A, Sigurðardottir S, Gulcher JR, Ward R, Stefánsson K (2000) mtDNA and the origin of the Icelanders: Deciphering signals of recent population history. American Journal of Human Genetics 66: 999-1016.

3. Als TD, Jorgensen TH, Borglum AD, Petersen PA, Mors O, et al. (2006) Highly discrepant proportions of female and male Scandinavian and British Isles ancestry within the isolated population of the Faroe Islands. Eur J Hum Genet 14: 497-504.

4. Helgason A, Hickey E, Goodacre S, Bosnes V, Stefansson K, et al. (2001) mtDNA and the Islands of the North Atlantic: Estimating the Proportions of Norse and Gaelic Ancestry. Am J Hum Genet 68: 723-737.

5. Goodacre S, Helgason A, Nicholson J, Southam L, Ferguson L, et al. (2005) Genetic evidence for a family-based Scandinavian settlement of Shetland and Orkney during the Viking periods. Heredity 95: 129-135.

6. McEvoy B, Richards M, Forster P, Bradley DG (2004) The Longue Duree of genetic ancestry: multiple genetic marker systems and Celtic origins on the Atlantic facade of Europe. Am J Hum Genet 75: 693-702.

7. Richards M, Macaulay V, Hickey E, Vega E, Sykes B, et al. (2000) Tracing european founder lineages in the near eastern mtDNA pool. Am J Hum Genet 67: 1251-1276.

8. Opdal SH, Rognum TO, Vege A, Stave AK, Dupuy BM, et al. (1998) Increased number of substitutions in the D-loop of mitochondrial DNA in the sudden infant death syndrome. Acta Paediatrica 87: 1039-1044.

9. Lappalainen T, Laitinen V, Salmela E, Andersen P, Huoponen K, et al. (2008) Migration Waves to the Baltic Sea Region. Ann Hum Genet.

10. Brandstatter A, Niederstatter H, Pavlic M, Grubwieser P, Parson W (2007) Generating population data for the EMPOP database - an overview of the mtDNA sequencing and data evaluation processes considering 273 Austrian control region sequences as example. Forensic Sci Int 166: 164-175.

11. Parson W, Parsons TJ, Scheithauer R, Holland MM (1998) Population data for 101 Austrian Caucasian mitochondrial DNA d-loop sequences: Application of mtDNA sequence analysis to a forensic case. International Journal of Legal Medicine 111: 124-132.

12. Baasner A, Schafer C, Junge A, Madea B (1998) Polymorphic sites in human mitochondrial DNA control region sequences: population data and maternal inheritance. Forensic Sci Int 98: 169-178.

13. Lutz S, Weisser HJ, Heizmann J, Pollak S (1998) Location and frequency of polymorphic positions in the mtDNA control region of individuals from Germany. International Journal of Legal Medicine 111: 67-77.

14. Richards M, Corte-Real H, Forster P, Macaulay V, Wilkinson-Herbots H, et al. (1996) Paleolithic and neolithic lineages in the European mitochondrial gene pool. American Journal of Human Genetics 59: 185-203.

15. Poetsch M, Wittig H, Krause D, Lignitz E (2003) Mitochondrial diversity of a northeast German population sample. Forensic Sci Int 137: 125-132.

16. Tetzlaff S, Brandstatter A, Wegener R, Parson W, Weirich V (2007) Mitochondrial DNA population data of HVS-I and HVS-II sequences from a northeast German sample. Forensic Sci Int 172: 218-224.

17. Dimo-Simonin N, Grange F, Taroni F, Brandt-Casadevall C, Mangin P (2000) Forensic evaluation of mtDNA in a population from south west Switzerland. International Journal of Legal Medicine 113: 89-97.

18. Pult I, Sajantila A, Simanainen J, Georgiev O, Schaffner W, et al. (1994) Mitochondrial-DNA sequences from Switzerland reveal striking homogeneity of European populations. Biological Chemistry Hoppe-Seyler 375: 837-840.

19. Jorde LB, Watkins WS, Bamshad MJ, Dixon ME, Ricker CE, et al. (2000) The distribution of human genetic diversity: a comparison of mitochondrial, autosomal, and Y-chromosome data. Am J Hum Genet 66: 979-988.

20. Brehm A, Pereira L, Kivisild T, Amorim A (2003) Mitochondrial portraits of the Madeira and Acores archipelagos witness different genetic pools of its settlers. Hum Genet 114: 77-86.

21. Dubut V, Chollet L, Murail P, Cartault F, Beraud-Colomb E, et al. (2004) mtDNA polymorphisms in five French groups: importance of regional sampling. Eur J Hum Genet 12: 293-300.

22. Richard C, Pennarun E, Kivisild T, Tambets K, Tolk HV, et al. (2007) An mtDNA perspective of French genetic variation. Ann Hum Biol 34: 68-79.

23. Rousselet F, Mangin P (1998) Mitochondrial DNA polymorphisms: A study of 50 French Caucasian individuals and application to forensic casework. International Journal of Legal Medicine 111: 292-298.

24. Falchi A, Giovannoni L, Calo CM, Piras IS, Moral P, et al. (2006) Genetic history of some western Mediterranean human isolates through mtDNA HVR1 polymorphisms. J Hum Genet 51: 9-14.

25. Achilli A, Olivieri A, Pala M, Metspalu E, Fornarino S, et al. (2007) Mitochondrial DNA variation of modern Tuscans supports the near eastern origin of Etruscans. Am J Hum Genet 80: 759-768.

26. Francalacci P, Bertranpetit J, Calafell F, Underhill PA (1996) Sequence diversity of the control region of mitochondrial-DNA in Tuscany and its implications for the peopling of Europe. American Journal of Physical Anthropology 100: 443-460.

27. Tagliabracci A, Turchi C, Buscemi L, Sassaroli C (2001) Polymorphism of the mitochondrial DNA control region in Italians. Int J Legal Med 114: 224-228.

28. Dirienzo A, Wilson AC (1991) Mitochondrial-Dna and the Dispersal of Early Humans. American Journal of Human Genetics 49: 459.

29. Cali F, Le Roux MG, D'Anna R, Flugy A, De Leo G, et al. (2001) MtDNA control region and RFLP data for Sicily and France. Int J Legal Med 114: 229-231.

30. Vanecek T, Vorel F, Sip M (2004) Mitochondrial DNA D-loop hypervariable regions: Czech population data. Int J Legal Med 118: 14-18.

31. Malyarchuk BA, Grzybowski T, Derenko MV, Czarny J, Wozniak M, et al. (2002) Mitochondrial DNA variability in Poles and Russians. Ann Hum Genet 66: 261-283.

32. Zupanic Pajnic I, Balazic J, Komel R (2004) Sequence polymorphism of the mitochondrial DNA control region in the Slovenian population. Int J Legal Med 118: 1-4.

33. Malyarchuk B, Derenko M, Grzybowski T, Lunkina A, Czarny J, et al. (2004) Differentiation of mitochondrial DNA and Y chromosomes in Russian populations. Hum Biol 76: 877-900.

34. Malyarchuk BA, Derenko MV (2001) Mitochondrial DNA variability in Russians and Ukrainians: implication to the origin of the Eastern Slavs. Ann Hum Genet 65: 63-78.

35. Calafell F, Underhill P, Tolun A, Angelicheva D, Kalaydjieva L (1996) From Asia to Europe - Mitochondrial-DNA sequence variability in Bulgarians and Turks. Annals of Human Genetics 60: 35-49.

36. Owens KN, Harvey-Blankenship M, King MC (2002) Genomic sequencing in the service of human rights. Int J Epidemiol 31: 53-58.

37. Rando JC, Pinto F, Gonzalez AM, Hernandez M, Larruga JM, et al. (1998) Mitochondrial DNA analysis of Northwest African populations reveals genetic exchanges with European, Near-Eastern, and sub-Saharan populations. Annals of Human Genetics 62: 531-550.

38. Rando JC, Cabrera VM, Larruga JM, Hernandez M, Gonzalez AM, et al. (1999) Phylogeographic patterns of mtDNA reflecting the colonization of the Canary Islands. Annals of Human Genetics 63: 413-428.

39. Abu-Amero KK, Gonzalez AM, Larruga JM, Bosley TM, Cabrera VM (2007) Eurasian and African mitochondrial DNA influences in the Saudi Arabian population. BMC Evol Biol 7: 32.

40. Kivisild T, Reidla M, Metspalu E, Rosa A, Brehm A, et al. (2004) Ethiopian mitochondrial DNA heritage: tracking gene flow across and around the gate of tears. Am J Hum Genet 75: 752-770.
